# Signalling mechanisms underlying doxorubicin and Nox2 NADPH oxidase‐induced cardiomyopathy: involvement of mitofusin‐2

**DOI:** 10.1111/bph.13773

**Published:** 2017-04-22

**Authors:** Declan McLaughlin, Youyou Zhao, Karla M O'Neill, Kevin S Edgar, Philip D Dunne, Anna M Kearney, David J Grieve, Barbara J McDermott

**Affiliations:** ^1^ Centre for Experimental Medicine, Wellcome‐Wolfson Building Queen's University Belfast Belfast UK; ^2^ Centre for Cancer Research and Cell Biology Queen's University Belfast Belfast UK

## Abstract

**Background and Purpose:**

The anthracycline doxorubicin (DOX), although successful as a first‐line cancer treatment, induces cardiotoxicity linked with increased production of myocardial ROS, with Nox2 NADPH oxidase‐derived superoxide reported to play a key role. The aim of this study was to identify novel mechanisms underlying development of cardiac remodelling/dysfunction further to DOX‐stimulated Nox2 activation.

**Experimental Approach:**

Nox2^−/−^ and wild‐type (WT) littermate mice were administered DOX (12 mg·kg^−1^ over 3 weeks) prior to study at 4 weeks. Detailed mechanisms were investigated in murine HL‐1 cardiomyocytes, employing a robust model of oxidative stress, gene silencing and pharmacological tools.

**Key Results:**

DOX‐induced cardiac dysfunction, cardiomyocyte remodelling, superoxide production and apoptosis in WT mice were attenuated in Nox2^−/−^ mice. Transcriptional analysis of left ventricular tissue identified 152 differentially regulated genes (using adjusted *P* < 0.1) in DOX‐treated Nox2^−/−^ versus WT mice, and network analysis highlighted ‘Cell death and survival’ as the biological function most significant to the dataset. The mitochondrial membrane protein, mitofusin‐2 (Mfn2), appeared as a strong candidate, with increased expression (1.5‐fold), confirmed by qPCR (1.3‐fold), matching clear published evidence of promotion of cardiomyocyte cell death. In HL‐1 cardiomyocytes, targeted siRNA knockdown of Nox2 decreased Mfn2 protein expression, but not *vice versa*. While inhibition of Nox2 activity along with DOX treatment attenuated its apoptotic and cytotoxic effects, reduced apoptosis after Mfn2 silencing reflected a sustained cytotoxic response and reduced cell viability.

**Conclusions and Implications:**

DOX‐induced and Nox2‐mediated up‐regulation of Mfn2, rather than contributing to cardiomyocyte dysfunction through apoptotic pathways, appears to promote a protective mechanism.

**Linked Articles:**

This article is part of a themed section on New Insights into Cardiotoxicity Caused by Chemotherapeutic Agents. To view the other articles in this section visit http://onlinelibrary.wiley.com/doi/10.1111/bph.v174.21/issuetoc

**Abbreviations**
AFC: glycyl‐phenylalanyl‐aminofluorocoumarin
DOX: doxorubicin
DPI: diphenyleneiodonium
HPRT: hypoxanthine phosphoribosyltransferase
HSP: heat shock protein
IVSD: interventricular septal thickness in diastole
IVSS: interventricular septal thickness in systole
LF2000: Lipofectamine™ 2000
L‐NAME: L‐N^G^‐nitroarginine methyl ester
LV: left ventricular
LVEDD: left ventricular end‐diastolic diameter
LVPWD: left ventricular posterior wall dimension in diastole
LVPWS: left ventricular posterior wall dimension in systole
LVESD: left ventricular end‐systolic diameter
Mfn2: mitofusin‐2
MTT: 3‐(4,5‐dimethylthiazol‐2‐yl)‐2,5‐diphenyltetrazolium
MV: mitral valve
PBMCs: peripheral blood mononuclear cells
PDK1: 3‐phosphoinositide dependent protein kinase 1
PGC‐1α: peroxisome activated receptor γ coactivator‐1α
R110: bis‐alanylalanyl‐phenylalanyl‐rhodamine 110
WT: wild‐type

## Introduction

Anthracycline drugs, such as doxorubicin (DOX), are effective anti‐tumour drugs commonly prescribed to treat haematological malignancies and solid tumours, but their use is severely limited by a dose‐dependent, cumulative and irrevocable cardiotoxicity. This is characterized by significant changes in cardiomyocyte biology and the extracellular matrix – the process of cardiac remodelling, which progresses to chamber dilatation, contractile dysfunction and chronic heart failure (Bloom *et al.,* 2016). Current therapeutic strategies for DOX cardiotoxicity include standard heart failure medications, such as β‐adrenoceptor antagonists, angiotensin receptor antagonists and angiotensin converting enzyme inhibitors, which can reduce the progression of early cardiotoxicity, although their efficacy in the longer term is limited (Cardinale *et al.,* 2015; Spallarossa *et al.,* 2016). Use of cardioprotective agents, such as desrazoxane, has shown benefits in certain patient groups, and so, modulation of redox mechanisms is considered a worthwhile tactic, although how to target these is not at all clear (Deidda *et al.,* 2016). Thus, further understanding the molecular phenotype and signalling mechanisms of DOX‐induced cardiotoxicity is fundamental to the development of effective preventive strategies, and thereby improved chemotherapy outcome.

Cardiomyocyte loss through cell death pathways is a customary paradigm to explain functional deficits in the heart, and there is ample experimental evidence to support DOX‐induced apoptosis, necrosis and autophagy, as reviewed by Carvalho *et al.* (2014). One of the major theories of the action of DOX is based on its interference with iron metabolism and generation of excess of ROS. However, although antioxidants, such as co‐enzyme Q10, N‐acetylcysteine and vitamins C and E, have been reported to exert cardioprotective effects in experimental models (Sterba *et al.,* 2013), the results of small randomized clinical trials have not shown clear benefit (van Dalen *et al.,* 2011; Vincent *et al.,* 2013). The lack of success of antioxidant therapeutic strategies is likely to demonstrate the complexity of redox reactions in biological tissues (Madamanchi and Runge, 2013), in which ROS are known to serve both physiological and maladaptive roles. It is likely, therefore, that selective targeting of particular sources of ROS or downstream effectors may represent a more viable approach. In addition to NOS signalling, ROS generated through NADPH oxidase play an essential role in cardiac pathophysiology, regulating major elements of cardiac remodelling, such as fibrosis and apoptosis (Grieve *et al.,* 2006; Gilleron *et al.,* 2009). There is accumulating evidence to support an important role for Nox2 NADPH oxidase in DOX‐induced cardiotoxicity, identified using Nox2‐deficient (Nox2^−/−^) mice (Wojnowski *et al.,* 2005; Deng *et al.,* 2007; Zhao *et al.,* 2010). Indeed, our group previously reported that DOX‐induced interstitial fibrosis, leukocyte infiltration, cardiomyocyte apoptosis and atrophy, and cardiac dysfunction were attenuated in Nox2^−/−^ mice (Zhao *et al.,* 2010).

A great deal of mechanistic work has been performed in both *in vivo* and *in vitro* models from which a complex picture of signalling pathways underlying DOX‐induced cardiotoxicity has emerged, in which cell death is balanced by intracellular survival signalling, linked to neuregulin/ErbB2 and Akt activation (Ghigo *et al.,* 2016). In promoting cell death, oxidative stress from ROS, including superoxide and peroxynitrite, cause activation of kinase pathways (MAPK 4/7, checkpoint kinase 2, stress‐activated protein kinase, JNK). Suppression of transcription factors, GATA‐4 (Kobayashi *et al*., 2010; Suzuki, 2011) and p300 (Poizat *et al.,* 2005), is also linked to regulation of cell survival. Induction of small heat shock proteins (e.g. HSP20, HSP21, HSP2, HSP70) can be either cardioprotective or detrimental in this setting (Liu *et al.,* 2007; Vedam *et al.,* 2010; Wang *et al.,* 2016). Other putative mechanisms include damage to nuclear DNA, disruption of sarcomeric protein synthesis (Ito *et al.,* 1990), accumulation of the tumour suppressor protein, p53 (Yoshida *et al.,* 2009) and a disturbance of energy metabolism (Tokarska‐Schlattner *et al.,* 2006). In mitochondria, increased ROS leads to Ca^2+^ overload, which triggers mitochondrial permeability transition, resulting in loss of membrane potential, swelling and outer membrane rupture, and consequent activation of caspases, release of cytochrome *c* and apoptosis.

Considering the strong evidence supporting a key role for Nox2‐derived ROS in DOX‐induced cardiotoxicity and the large number of possible signalling pathways identified, the primary purpose of this investigation was to highlight relevant Nox2‐regulated genes and potential networks in this setting. Use of mRNA microarray technology (Kuhn *et al.,* 2004) and the Nox2^−/−^ mouse model (Zhao *et al.,* 2010) was considered a suitable approach. Having identified the mitochondrial membrane protein, mitofusin‐2 (Mfn2), as a strong candidate, the hypothesis that up‐regulated Mfn2 contributes to cardiomyocyte death processes induced by DOX was tested.

## Methods

### Experimental model

#### Animals

Mouse models incorporating genetic disruption underpin mechanistic evaluation of the contribution of particular signalling pathways, and here, we have used Nox2^−/−^ mice to investigate the influence of ROS production and downstream effectors in DOX‐induced cardiotoxicity. Nox2^−/−^ mice on a C57BL/6J background (Pollock *et al.,* 1995), originally obtained from Jackson Laboratories (Bar Harbour, USA), were bred from an established colony at Queen's University Belfast. Animal studies are reported in compliance with the ARRIVE guidelines (Kilkenny *et al.*, 2010; McGrath and Lilley, 2015). All experimental procedures were carried out in accordance with the Home Office Guidance on the Operation of the Animals (Scientific Procedures) Act 1986, published by Her Majesty's Stationary Office, London, and approved by the Queen's University Belfast Animal Welfare and Ethical Review Body (PPL2714). All mice were housed in the Queen's University Belfast Biological Services Unit under controlled conditions (12 h light–dark cycle, 21°C) in standard caging, typically together with three to five littermates.

#### DOX administration

Male Nox2^−/−^ and wild‐type (WT) littermate controls (8–10 weeks old, 25–28 g) were randomized prior to light anaesthesia with 1.5% isofluorane for administration of a cumulative dose of 12 mg·kg^−1^ DOX or saline control by three weekly injections (4 mg·kg^−1^ i.p. at 0, 7 and 14 days). All subsequent analyses were performed 4 weeks after the first injection. Selection of the 4 week time point was based on the progression of DOX‐induced cardiac contractile dysfunction, by which time a maximum decrease in percentage fractional shortening was achieved (Supporting Information Fig. S1).

### Assessment of cardiac remodelling

#### Echocardiography

Mice were anaesthetised with 1.5% isofluorane/oxygen, placed on a warming pad and imaged in the supine position using a Vevo770® ultrasound system with high‐frequency 45 MHz RMV707B scan head (VisualSonics Inc.). M‐mode parasternal short‐axis scans at the level of the papillary muscles were used to quantify LV wall thickness (interventricular septal thickness in diastole, IVSD; interventricular septal thickness in systole, IVSS; left ventricular posterior wall dimension in diastole, LVPWD; left ventricular posterior wall dimension in systole, LVPWS) and LV end‐diastolic and end‐systolic diameters (LVEDD, LVESD) from which percentage fractional shortening was calculated (LVEDD‐LVESD)/LVEDD*100). Pulse‐wave Doppler was used to quantify mitral valve (MV) flow, expressed as E/A ratio.

#### Morphometric assessment

The animals were killed by an overdose of sodium pentobarbitone (Euthanal®; 200 mg·kg^−1^, i.p.), before the heart was excised and ventricles divided. Left ventricular (LV) weight was taken and indexed to tibial length. LV tissue was cut into transverse sections, which were flash frozen in liquid nitrogen prior to storage at −80°C or immersed in 10% (v^.^v^‐1^) neutral‐buffered formalin for histological analyses.

#### Histological analyses

Fixed LV tissue was dehydrated using graded ethanol solutions (30–100%, v^.^v^‐1^) and xylene, before being embedded in paraffin wax and cut into thin sections (5 μm). Standard haematoxylin and eosin staining was used to quantify cardiomyocyte cross‐sectional area; only cells with centrally located nuclei were analysed. Cardiomyocyte apoptosis was assessed by TUNEL staining (Roche Diagnostics). TUNEL‐positive nuclei were expressed as % total nuclei stained with DAPI (1:1000; Invitrogen) in the same sections. For analysis of both cardiomyocyte cross‐sectional area and TUNEL staining, LV sections were visualized by fluorescence microscopy and quantified using blinded digital image analysis (NIS‐Elements). Each slide contained at least four sections, which were each divided into four microscopic areas, from which five separate cells were measured, such that for one animal, a total of 80 cells were analysed.

### NADPH oxidase activity

LV tissue samples stored at −80°C were homogenized in lysis buffer (20 mM HEPES, 4 mM EGTA, 1 mM DTT, 6.25 μL·mL^−1^ protease inhibitor cocktail; 1 mL·100 mg^−1^), sonicated and membrane fractions prepared from supernatants by centrifugation at 12 000 *g* for 60 min. In samples diluted to a concentration of 1 mg protein·mL^−1^, NADPH‐dependent superoxide production was measured by lucigenin (5 μM)‐enhanced chemiluminescence at 37°C for 30 min (Zhao *et al.,* 2010). Potential sources of superoxide were assessed in experiments including the following: (a) tiron (20 mM), cell‐permeable superoxide scavenger; (b) diphenyleneiodonium (DPI, 10 μM), inhibitor of NADPH oxidase and other flavoproteins; (c) L‐N^G^‐nitroarginine methyl ester (L‐NAME, 1 mM), inhibitor of superoxide production by dysfunctional NOS; (d) oxypurinol (100 μM), xanthine oxidase inhibitor and (e) rotenone (10 μM), which inhibits the mitochondrial electron transport chain.

### Gene expression analysis using real‐time RT‐PCR

Total RNA was extracted from LV homogenate using TRI‐Reagent (Sigma‐Aldrich). RNA concentration was measured at 260 nm using a Thermo Scientific NanoDrop™ 1000 spectrophotometer and purity determined as the 260:280 nm ratio; samples with readings 1.8–2.0 were considered of acceptable purity and taken forward at equal concentrations for reverse transcription using a High‐Capacity cDNA Reverse Transcription Kit (Applied Biosystems). Quantification of mRNA expression was performed by real‐time RT‐PCR using Power SYBR®Green on an ABI 7300 Real‐Time PCR System (Applied Biosystems) using standard procedures. Primer Express Software (Applied Biosystems) was used to generate mouse‐specific primer pairs (Supporting Information Table S1), which were custom synthesized by Invitrogen. Pre‐designed and validated inventoried TaqMan® Gene Expression Assays with a FAM™‐labelled probe (Applied Biosystems) were also employed for quantification of Nox2 mRNA expression. Difference in threshold cycle (Ct) for a particular PCR product from that of an endogenous control, β‐actin (ΔCt), was calculated, and data expressed in each experiment relative to the control group (ΔΔCt).

### Western blotting

LV protein was extracted by homogenization with ice‐cold RIPA buffer, as previously described (Zhao *et al.,* 2010), and 20 μg loaded onto a 10% SDS‐PAGE gel before blotting on a PVDF membrane (Immobilon‐FL; Millipore). Membranes were incubated overnight at 4°C with a rabbit monoclonal antibody against Nox2 (1:1000, Abcam ab129068) using hypoxanthine phosphoribosyltransferase (HPRT) antibody (1:10 000, ab109021 Abcam) as a loading control. This was followed by incubation with horseradish peroxidase‐labelled goat anti‐rabbit secondary antibody (1:10 000 Cell Signalling Technology #7074P2) for 60 min at room temperature, before the membrane was developed in a darkroom using Immobilon Western Chemiluminescent HRP Substrate (Millipore), scanned and quantified by densitometry (ImageJ). Variations in band density are expressed as fold changes compared with the HPRT control.

### Gene expression profiling

LV tissue was obtained from three male Nox2^−/−^ and three WT littermate controls, treated with DOX, as above. Before the animals were killed at 4 weeks, contractile dysfunction was confirmed in WT but not Nox2^−/−^ mice, demonstrating that these animals developed a similar cardiotoxicity to those in the experimental cohorts. LV tissue was homogenized, total RNA extracted and quantitative/qualitative analyses were performed as above to check adequate purity and then diluted to a pre‐determined concentration of 100 ng·μL^−1^ RNA in 20 μL diethylpyrocarbonate‐treated water. Microarray analysis including quality control assessment was conducted by Cambridge Genomics Services.

#### Quality control

Initially, the RNA integrity number for all gene array samples was shown to have a ratio of 2:1, confirming that no significant degradation of RNA product had occurred. RNA samples were amplified using the Ambion Illumina® TotalPrep RNA Amplification Kit, incorporating biotin for hybridization with an Illumina MouseWG‐6 v2.0 array. After hybridization, the bead array was washed, stained and scanned (Illumina Iscan), and files were loaded into Genome Studio (Illumina) to assess performance of the analysis with the following checks included: (a) hybridization – three concentrations of Cy3‐labelled oligonucleotides, each with perfect matches to control probes on the bead array; (b) low stringency – two sample independent oligonucleotides, a mismatch (mm2) probe and a perfect match (pm) probe, both compared with a control oligonucleotide; (c) high stringency – one probe corresponding to a Cy3‐labelled oligonucleotide target, probe‐target pairing having a high GC base pair content; (d) biotin staining – a sample‐independent specific labelled oligonucleotide matched and bound to a probe on the microarray; (e) background – oligonucleotides of a random sequence, selected to have no corresponding targets in the genome, the mean signal of these oligonucleotide‐probe interactions representing the imaging system background as well as any signal resulting from non‐specific binding of dye or cross‐hybridization; (f) signal intensity – housekeeping genes and ‘all gene’ controls (Illumina) to check for sample degradation, including two oligonucleotides per gene matched with targets on the bead array.

To assess biological correlation within and between experimental groups, data were exported to the software package *R* and analysed using the embedded *lumi* package from Bioconductor (Du *et al.,* 2008). Data were initially filtered to remove any probes not detected at least once in the array readout. Values close to background were also deleted to prevent these genes adding noise to the data. A threshold for selection was set at *P* < 0.01, and this criterion was applied in such a way that for a gene to be detected, it had to be present in all the samples. The new data set was then re‐analysed employing less stringent parameters, whereby genes were selected based on the presence of at least one of the arrays or samples analysed. Probes that met these criteria were transformed using a variance stabilization algorithm (Lin *et al.,* 2008), similar to a log2 transformation, prior to quantile normalization. Using correlation analysis, samples were plotted against each other, such that those with a high correlation value (close to 1) show similar expression profiles, while those with a high negative correlation value (i.e. close to −1) show different expression profiles. As the aim of this study was to highlight and subsequently validate the biologically relevant signalling pathways underpinning differential DOX‐induced cardiac remodelling in WT versus Nox2^−/−^ mice, an established holistic pathway approach using low stringency selection of genes of interest was followed. This prevents exclusion of potentially important gene changes, which may become more relevant when viewed in the context of altered expression of an entire signalling pathway (see below).

Hierarchical clustering of samples and principal component analysis were performed in our laboratory using Partek Genomics Suite with default parameters. Data matrices were standardized to the median value of probe sets expression. Standardization of the data allows for comparison of expression levels for different probe sets. Following standardization, 2‐dimensional hierarchical clustering was performed (samples x probe sets/genes). Euclidean distance was used to calculate the distance matrix, a multidimensional matrix representing the distance from each data point (probe set‐sample pair) to all the other data points. Ward's linkage method was subsequently applied to join samples and genes together, with the minimum variance, to find compact clusters based on the calculated distance matrix.

#### Identification of genes differentially expressed in WT versus Nox2^−/−^ hearts

The normalized data set was analysed using the *limma* package from Bioconductor to generate LogFC (log_2_ fold change) values for each probe (gene ID), each with an associated *P* value from application of a modified *t*‐test. The False Discovery Rate adjusted *P* value, controlling for the number of false positives in tests that produce a significant result, was used as the primary filtering parameter.

#### Network analysis

In order to identify potential signalling pathways regulated by differentially expressed genes, Ingenuity Pathway Analysis software incorporating the Ingenuity Knowledge Base, curated from primary literature, as well as public and third‐party databases, was used to analyse the normalized dataset. An adjusted *P* value of <0.1 was applied to include a sufficient number of genes for generation of candidate molecules and pathways.

#### Gene expression by real‐time RT‐PCR

mRNA analysis of the most relevant genes was performed in LV tissue from all experimental groups, and primer sequences are shown in Supporting Information Table S1.

### HL‐1 cardiomyocyte model

HL‐1 cardiomyocytes were a gift from Dr William C. Claycomb (Louisiana State University Health Science Centre, New Orleans). Cells were grown in T75 flasks coated with gelatin (0.02%) plus fibronectin (12.5 mg·mL^−1^) and were maintained in Claycomb medium (Sigma‐Aldrich), supplemented with 10% FBS, 2 mM L‐glutamine, 10 mM penicillin–streptomycin (Life Technologies) and 10 mM noradrenaline (Sigma‐Aldrich) at 37°C and 5% CO_2_. The culture medium was changed approximately every 48 h, and cells were passaged upon reaching 80–90% confluency.

#### Acute DOX stimulation of HL‐1 cardiomyocytes

For different experiments, cells were seeded in 12‐ or 24‐ or 96‐well plates (Nunc) at a density of 400 000 or 200 000 or 100 000 cells per well respectively. After 24 h, cells were washed with PBS to remove cellular debris and then treated with normal supplemented Claycomb medium (as above) as a control or with DOX (0.5, 5.0 or 50 μM) for 3 or 6 h.

#### Characterization of HL‐1 model (protein expression, ROS production)

For western blotting, cell extracts were prepared by addition of ice cold RIPA buffer (300 μL per well of a 12‐well plate; 1 mL per T25 or T75 cell culture flask) containing protease inhibitor cocktail (200 μL·40 mL^−1^). Cells were scraped from the adherent layer, the cell suspension was centrifuged at 12000 *g* for 15–20 min at 4°C and the supernatant analysed for protein before western blot analysis was performed as described above using primary antibodies detecting Nox2 (1:1000, Abcam ab129068) and Mfn2 (1:1000 Abcam ab50838). For measurement of ROS production, homogenates were prepared by probe sonication of the whole cell preparation on ice for 20 s and lucigenin‐enhanced chemiluminescence determined as above.

### Transfection of HL‐1 cardiomyocytes

HL‐1 cardiomyocytes were plated on the day before transfection in the absence of noradrenaline or antibiotic. Cells were then transfected using (1) Lipofectamine™ 2000 (LF2000, Life Technologies) with Silencer Select® siRNAs (Ambion, Life Technologies) against Mfn2 (4390771‐s100687) or Nox2 (4390771‐s64650), together with a Universal Negative Control (4390844) or (2) Dharmafect 1 Transfection Reagent (Dharmacon) along with SMARTpool ON‐TARGETplus siRNA against Mfn2 (L‐046303‐00‐0005) or Nox2 (L‐058659‐00‐000), both according to the manufacturer's instructions and following a Claycomb modified protocol. Briefly, for each well of a 24‐well plate, LF2000 (5 μL) was diluted in DMEM (100 μL) without serum or antibiotics and incubated for 5 min at room temperature. To this was added siRNA (200pM) in DMEM (100 μL) followed by incubation for 20 min before addition of noradrenaline/antibiotic‐free Claycomb medium containing 12.5% serum (0.8 mL). Culture medium was removed from each well, replaced with transfection medium and incubated at 37°C in a humidified CO_2_ incubator for 18 h, at which time a further 1 mL of noradrenaline/antibiotic‐free media was added. After 24 h, transfection medium was replaced with noradrenaline‐free supplemented Claycomb medium, without or with DOX as detailed in experiments below. Knockdown of targeted genes was quantified using western blotting methods as previously outlined.

### Measurement of cell death processes in HL‐1 cardiomyocytes

The ApoTox‐Glo™ Triplex assay (Promega) combines assessment of cell viability, cytotoxicity and caspase activation events within a single assay well. The first step simultaneously measures live and dead‐cell protease activities, using the fluorogenic substrates, glycyl‐phenylalanyl‐aminofluorocoumarin (AFC) and bis‐alanylalanyl‐phenylalanyl‐rhodamine 110 (R110) respectively. In the subsequent step utilizing the Caspase‐Glo® Assay Technology, a caspase‐3/7 substrate (tetrapeptide sequence DEVD) is added in a reagent optimized for caspase activity and a luminescent signal was generated by the addition of luciferase.

After treatments under control conditions or with DOX (5 μM) for 24 h, with or without pre‐exposure to transfection or siRNA agents, the AFC/R110 reagent (100 μL) was added to each well of a 24‐well plate, in parallel with no cell control wells. The plate was wrapped in aluminium foil and incubated at 37°C for 90 min. Fluorescence was measured using a TECAN Safire plate reader at excitation/emission wavelengths of 400/505 and 480/520 nm for measurement of AFC and R110 respectively. Then caspase 3/7 substrate (50 μL) was added to the wells and the plate incubated at room temperature for 1 h. The solution in each well was mixed, and the cell suspension decanted in 50 μL amounts, in quadruplicate, into black, opaque‐walled 96‐well plates and luminescence measured using a Berthold Tristar LB941 Multimode Reader luminometer. In all cases, background was accounted for by subtraction of the no cell control readings.

In a second set of experiments, HL1 cells were cultured in noradrenaline/antibiotic‐free medium for 24 h before being reverse transfected to deplete Mfn2 and then seeded onto a 96‐well plate using Dharmafect 1 and 100 nM ON‐TARGET plus SMARTpool Mfn2 siRNA or a matched concentration of non‐targeting siRNA (GE Healthcare Dharmacon Inc.). Medium was replaced with fresh complete media (containing noradrenaline but no antibiotics) 24 h post‐transfection. Cells were then treated with DOX (3 μM) 48 h post‐transfection (for 24 h). Endpoint measurements included caspase 3/7 activity (Caspase‐Glo® 3/7 Assay, Promega) performed according to manufacturer's instructions and cell viability assessed in each condition using 3‐(4,5‐dimethylthiazol‐2‐yl)‐2,5‐diphenyltetrazolium (MTT, 0.5 g·L^−1^). After removal of medium, DMSO (100 μL) was added to each well, incubated at 37°C for 15 min and absorbance read at 570 nm. An additional readout of caspase‐3‐mediated apoptosis was obtained by assessing cleaved PARP‐1 using a Pierce Colorimetric In‐Cell elisa Kit (Thermo Fisher) supplied with a whole‐cell stain (Janus Green).

### Inhibition of NADPH oxidase using VAS2870

HL‐1 cardiomyocytes in 24‐well plates were pretreated with the pan‐NADPH oxidase inhibitor, VAS2870 (10, 50 and 100 μM in 500 μL fresh culture medium), for 30 min. A further 500 μL culture medium containing 10 μM DOX and the inhibitor was added to each well, giving a final concentration of DOX of 5 μM. Cells were incubated for 24 h at 37°C prior to ApoTox‐Glo™ Triplex assay as above.

### Nomenclature of targets and ligands

Key protein targets and ligands in this article are hyperlinked to corresponding entries in http://www.guidetopharmacology.org, the common portal for data from the IUPHAR/BPS Guide to PHARMACOLOGY (Southan *et al.*, 2016), and are permanently archived in the Concise Guide to PHARMACOLOGY 2015/16 (Alexander *et al.*, 2015).

### Statistical analysis

Numbers of experiments in the four groups (control or DOX‐treated WT and Nox2^−/−^ mice) are based on data for coefficients of variation of relevant endpoints in LV tissue analysis measured in previous studies (Zhao *et al.,* 2010) and power calculations to allow detection of a 30% difference between groups with <5% false negative error. *In vivo* analysis of cardiac structure and function was performed in all of the cohorts, to include most of the animals in the study. For gene expression profiling, three biological replicates were considered adequate to detect sufficient differences between the two samples (LV tissue from DOX‐treated WT and Nox2^−/−^ mice) to enable meaningful pathway analysis. HL‐1 cardiomyocyte studies were performed using six to eight preparations for each endpoint, but data for a whole experiment were discarded if on occasion a response to DOX was not obtained. Data and statistical analysis comply with the recommendations on experimental design and analysis in pharmacology (Curtis *et al.,* 2015).

Data are expressed as mean ± SEM of *n* animals or tissue samples or cell preparations; values of *n* and number of technical replicates, if performed, are given in Figure and Table legends. Where replicates were conducted (Figures 8B and 9), these values were averaged to provide a single value contributing to the dataset. According to the design of the experiment, data were analysed using GraphPad Prism (Version 7.02) after application of the Brown‐Forsythe test to examine homogeneity of variance using a parametric one‐ or two‐factor ANOVA followed *post hoc*, when indicated for a particular factor (when F achieved *P* < 0.05), by a Bonferroni multiple comparison test for *n* > 2 group comparisons or if *n* = 2, a paired or unpaired Student's *t*‐test. When data have been expressed as fold change for comparison purposes of readouts with different baselines, the Kruskal–Wallis test followed by Dunn's multiple comparison test was applied. In all cases, *P* < 0.05 was considered to indicate statistical significance. Microarray data were analysed statistically as described in the relevant section.

## Results

### Effects of DOX on cardiac function, LV remodelling and Nox2‐dependent superoxide production

Echocardiography data taken from short axis M‐mode recordings (Table 1) indicated that heart rate, LV wall thickness (IVSD, IVSS, LVPWD, LVPWS) and chamber dimensions (LVEDD, LVESD) were unaltered comparing WT and Nox2^−/−^ mice with or without DOX treatment. However, LV systolic function, as measured by fractional shortening, was decreased by DOX in WT but not in Nox2^−/−^ mice. Similarly, diastolic dysfunction, quantified by a reduced MV E/A ratio versus that in controls, was evident in DOX‐treated WT but not in Nox2^−/−^ mice.

**Table 1:** Effects of DOX on cardiac structure and function

	WT control	WT DOX	Nox2^−/−^ Control	Nox2^−/−^ DOX
HR (beats·min^‐1^)	429 ± 10 (23)	449 ± 17 (24)	406 ± 13 (23)	436 ± 23 (29)
IVSD (mm)	0.80 ± 0.02 (23)	0.78 ± 0.02 (24)	0.84 ± 0.03 (23)	0.77 ± 0.02 (29)
IVSS (mm)	1.18 ± 0.06 (23)	1.13 ± 0.07 (24)	1.18 ± 0.15 (23)	1.16 ± 0.06 (29)
LVPWD (mm)	0.81 ± 0.03 (23)	0.79 ± 0.04 (24)	0.76 ± 0.02 (23)	0.94 ± 0.06 (29)
LVPWS (mm)	1.13 ± 0.07 (23)	1.07 ± 0.05 (24)	1.08 ± 0.07 (23)	1.28 ± 0.06 (29)
LVEDD (mm)	4.21 ± 0.05 (23)	4.21 ± 0.05 (24)	4.15 ± 0.06 (23)	4.06 ± 0.05 (29)
LVESD (mm)	2.91 ± 0.08 (23)	2.92 ± 0.07 (24)	3.02 ± 0.08 (23)	2.77 ± 0.08 (29)
Fractional shortening (%)	30.50 ± 1.42 (10)	26.06 ± 0.93* (16)	30.27 ± 1.67 (10)	28.91 ± 0.85 (17)
MV E/A	1.76 ± 0.07 (19)	1.44 ± 0.05* (17)	1.53 ± 0.10 (17)	1.63 ± 0.08 (25)
TL (mm)	18.36 ± 0.28 (19)	18.39 ± 0.28 (17)	18.32 ± 0.29 (22)	18.06 ± 0.22 (23)
LV/TL (mg·mm^−1^)	6.01 ± 0.30 (19)	5.14 ± 0.18* (17)	6.08 ± 0.18 (22)	5.73 ± 0.16 (23)
LV cardiomyocyte cross‐sectional area (μm^2^)	455.7 ± 25.6 (5)	347.5 ± 7.0* (5)	386.6 ± 1.3 (5)	394.7 ± 10.4 (5)

Electrocardiographic measurements were taken from short‐axis M‐mode recordings (HR, heart rate), and MV E/A ratio assessed by pulse‐wave Doppler flow. LV mass/tibial length (TL) ratio was assessed and myocyte cross‐sectional area measured after haematoxylin and eosin staining. Data are shown as mean ± SEM (*n*, number of animals) and analysis performed by two‐factor ANOVA, followed by Student's unpaired *t*‐test, as indicated.

* *P* < 0.05 versus WT Control.

**Figure 1:**
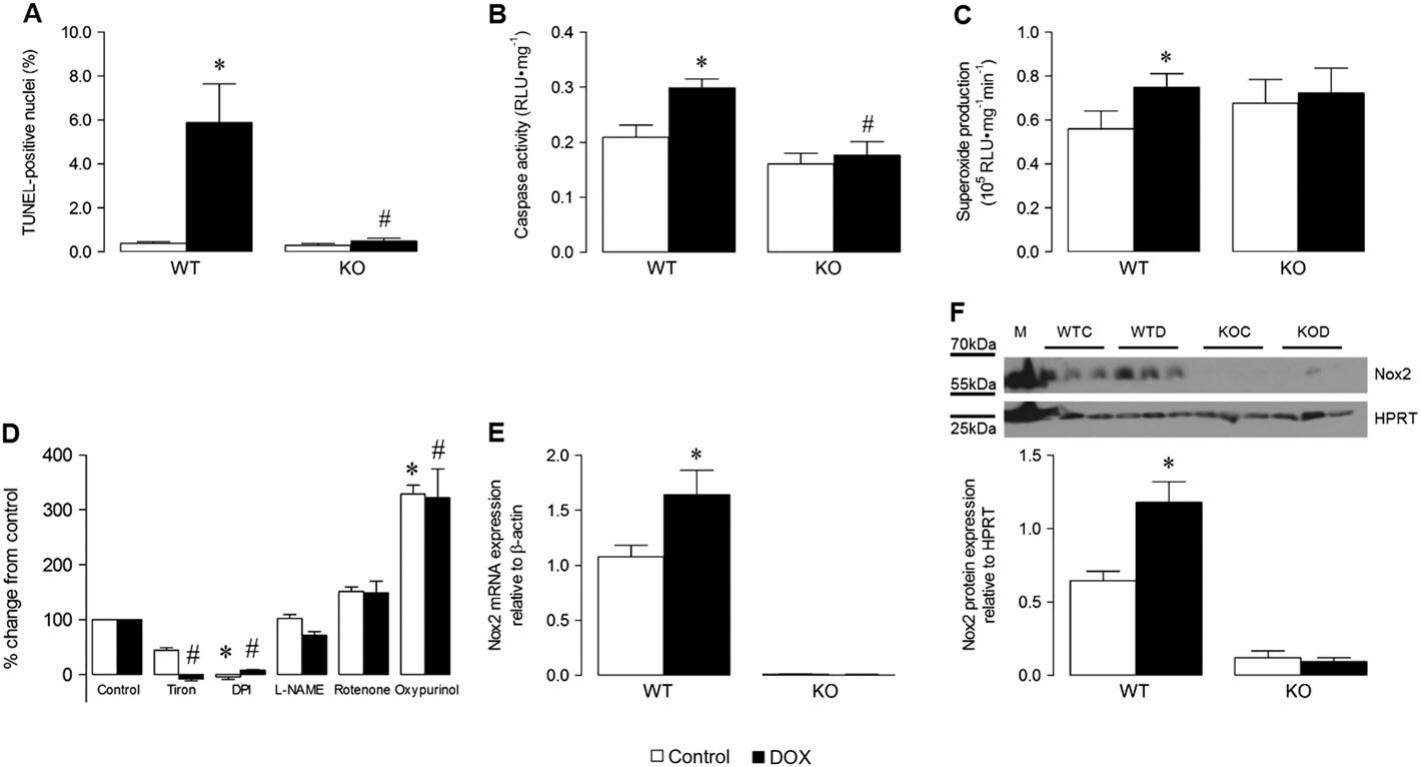
Effects of DOX on cardiomyocyte apoptosis, LV superoxide production and Nox2 mRNA and protein expression in WT and Nox2^−/−^ (KO) mice. (A) TUNEL‐positive cardiomyocyte nuclei in LV sections quantified by digital image analysis (*n* = 6, 80 cells in each). (B) Caspase 3/7 activity in LV tissue, expressed as relative light units (RLU; *n* = 5). (C) NADPH‐dependent superoxide production analysed in LV membrane fractions by lucigenin‐enhanced chemiluminescence (*n* = 6). (D) Effects of selective ROS inhibitors on superoxide production in WT samples (*n* = 6). (E) Nox2 mRNA expression assessed in LV tissue by real‐time TaqMan RT‐PCR (*n* = 15‐WT, *n* = 9‐KO). (F) Representative western blot of Nox2 protein expression in LV homogenate (M, MCF‐7 cell lysate positive control; WTC, WT control; WTD, WT DOX‐treated; KOC, KO control; KOD, KO DOX‐treated; HPRT, hypoxanthine‐guanine phosphoribosyltransferase endogenous control) and its quantification (*n* = 6). Data are shown as mean ± SEM and analyses performed using a two‐factor ANOVA followed by Student's unpaired *t*‐test (A–C) or Kruskal–Wallis test with Dunn's *post hoc* test for Control or DOX (D) or Student's unpaired *t*‐test with Walsh's correction to account for unequal variances (E, F). *P < 0.05 versus WT Control; ^#^ *P* < 0.05 versus WT DOX.

Examination of heart weight normalized to tibial length, which did not alter across groups, showed that DOX decreased LV mass in WT mice (Table 1), consistent with a cachectic effect, which was unaltered in Nox2^−/−^ DOX‐treated animals, compared with untreated control values. Histological analysis of cardiomyocyte cross‐sectional area confirmed that the observed action of DOX on LV mass was due to a specific effect on the cardiomyocyte; compared with control values, this was reduced in WT, but not in Nox2^−/−^ DOX‐treated animals, indicating that DOX‐induced cardiomyocyte atrophy is dependent upon Nox2 NADPH oxidase. Similarly, cardiomyocyte apoptosis was clearly exacerbated by DOX in LV sections from WT mice, in which the percentage of TUNEL‐positive cells increased approximately 10‐fold versus control, but this increase was considerably less (twofold) in Nox2^−/−^ mice (Figure 1A). Furthermore, the effect of DOX to increase activity of caspase 3/7 (key effector enzymes in the apoptotic process) in LV tissue from WT mice was largely attenuated in Nox2^−/−^ mice (Figure 1B), confirming that DOX‐induced cardiomyocyte apoptosis also appears to be Nox2‐dependent.

As expected, a DOX‐induced increase in LV NADPH‐dependent superoxide production was observed in WT but not in Nox2^−/−^ mice (Figure 1C), supporting the idea that Nox2‐dependent superoxide generation plays an important role in DOX cardiotoxicity. Notably, superoxide generation was inhibited by the superoxide scavenger, Tiron, and the flavoprotein inhibitor, DPI, but not by the NOS inhibitor, L‐NAME, the mitochondrial inhibitor, rotenone, or the xanthine oxidase inhibitor, oxypurinol (Figure 1D), suggesting that the observed signal particularly reflects NADPH oxidase‐derived superoxide. This finding is consistent with increased expression of Nox2 mRNA (Figure 1E) and protein (Figure 1F) in LV tissue from WT DOX‐treated animals versus controls. No differences in LV mRNA expression of NOS isoenzymes (neuronal NOS, inducible NOS, endothelial NOS), superoxide dismutases (SOD1, SOD2), glutaredoxin 1 and 2, catalase and glutathione peroxidase 1 were observed between DOX‐treated WT and Nox2^−/−^ mice (Supporting Information Figure S2).

### Microarray profiling of LV tissue and selection of candidate gene

**Figure 2:**
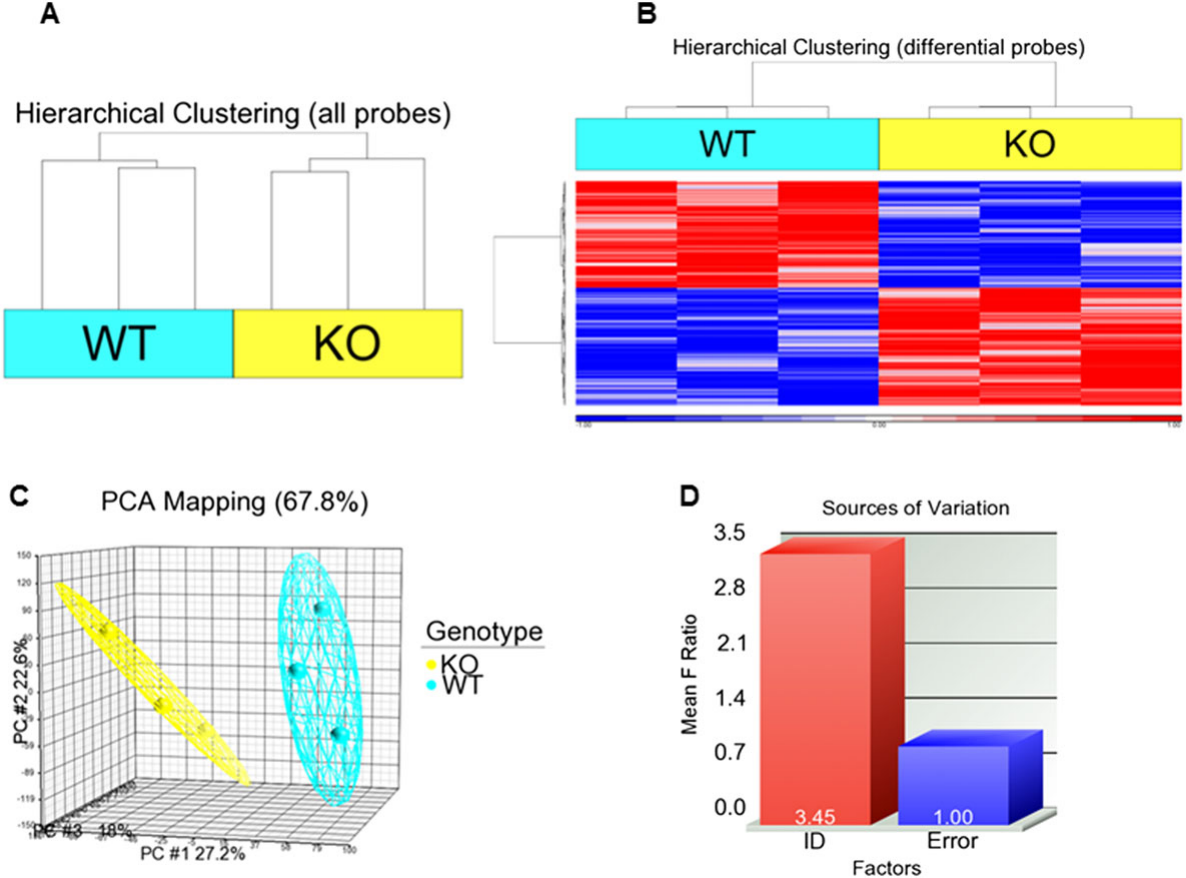
Microarray profiling of LV sample replicates from WT and Nox2^−/−^ (KO) DOX‐treated mice. (A) Unsupervised hierarchical clustering, (B) Heatmap representation of differentially expressed genes generated, (C) Principal component analysis and (D) Signal to noise ratio for tissue samples (ID) compared with background, generated using Partek Genomics Suite, using three mice in each group.

Samples of LV tissue from DOX‐treated WT and Nox2^−/−^ mice analysed using an Illumina MouseWG‐6 v2.0 microarray showed acceptable read‐outs in quality control assessment (Supporting Information Fig. S3). Specifically, low, medium and high levels of hybridization corresponded to signal intensities, low and high stringency controls performed appropriately, successful secondary biotin staining was demonstrated and the signal for the negative (background) control was low. Furthermore, as expected, the signal level of housekeeping genes was high in comparison with the signal for ‘all genes’. Using unsupervised hierarchical clustering, the six samples divided into two distinct groups based on mouse genotype (Figure 2A), with the heatmap representing differentially expressed genes (Figure 2B). A similar separation was observed by principal component analysis (Figure 2C), whilst the genotype label accounted for 3.45 times more variation above any background noise (error) even before filtering, providing confidence in the arrays (Figure 2D). The number of genes selected for normalization using the described filtering criteria was 16 825 from the original number of 45 281(43%), and normalized data from both WT and Nox2^−/−^ samples demonstrated good correlation (*r* ≥ 0.99) between biological replicates (Supporting Information Fig. S4).

**Figure 3:**
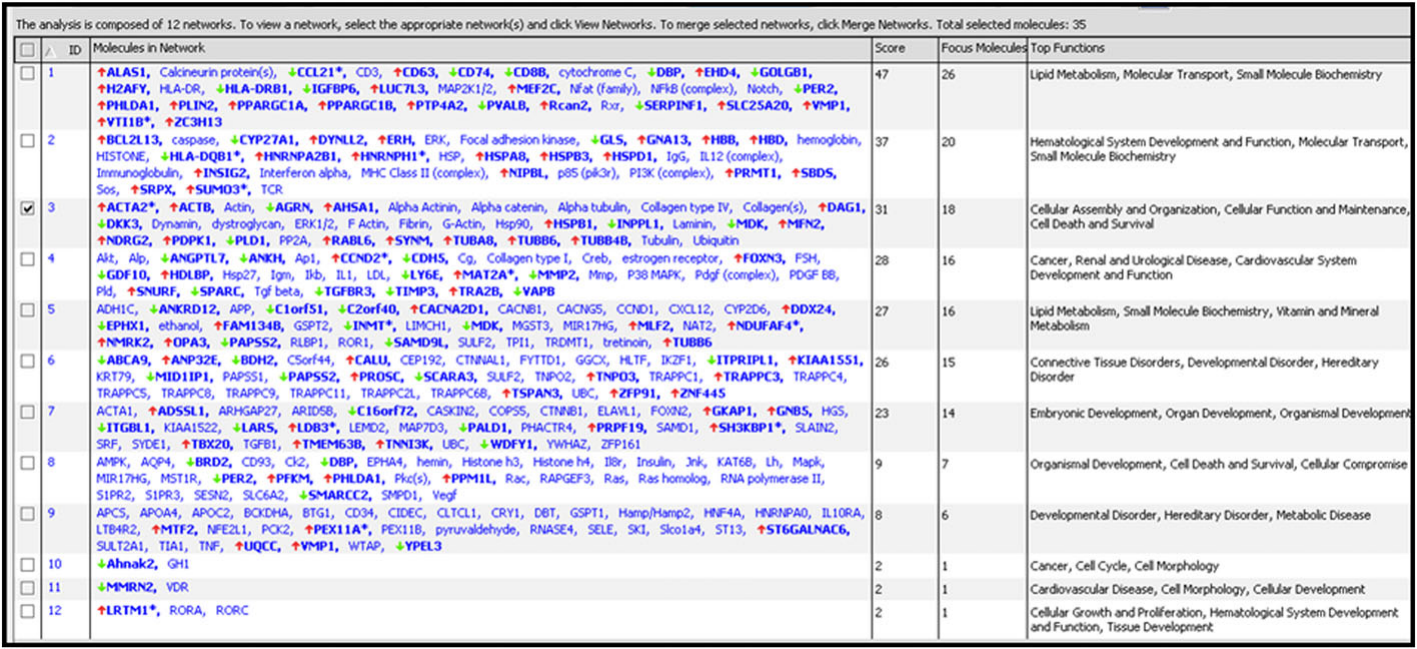
Network analysis of genes differentially regulated in LV tissue from WT versus Nox2^−/−^ DOX‐treated mice. A total of 152 genes (using cut‐off of adjusted *P* < 0.1) were analysed using Ingenuity Pathway Analysis software. Expression of genes in bold are significantly differentially regulated between groups (red arrows indicate up‐regulation and green arrows down‐regulation); genes not in bold are implicated in the networks but are not differentially regulated.

**Figure 4:**
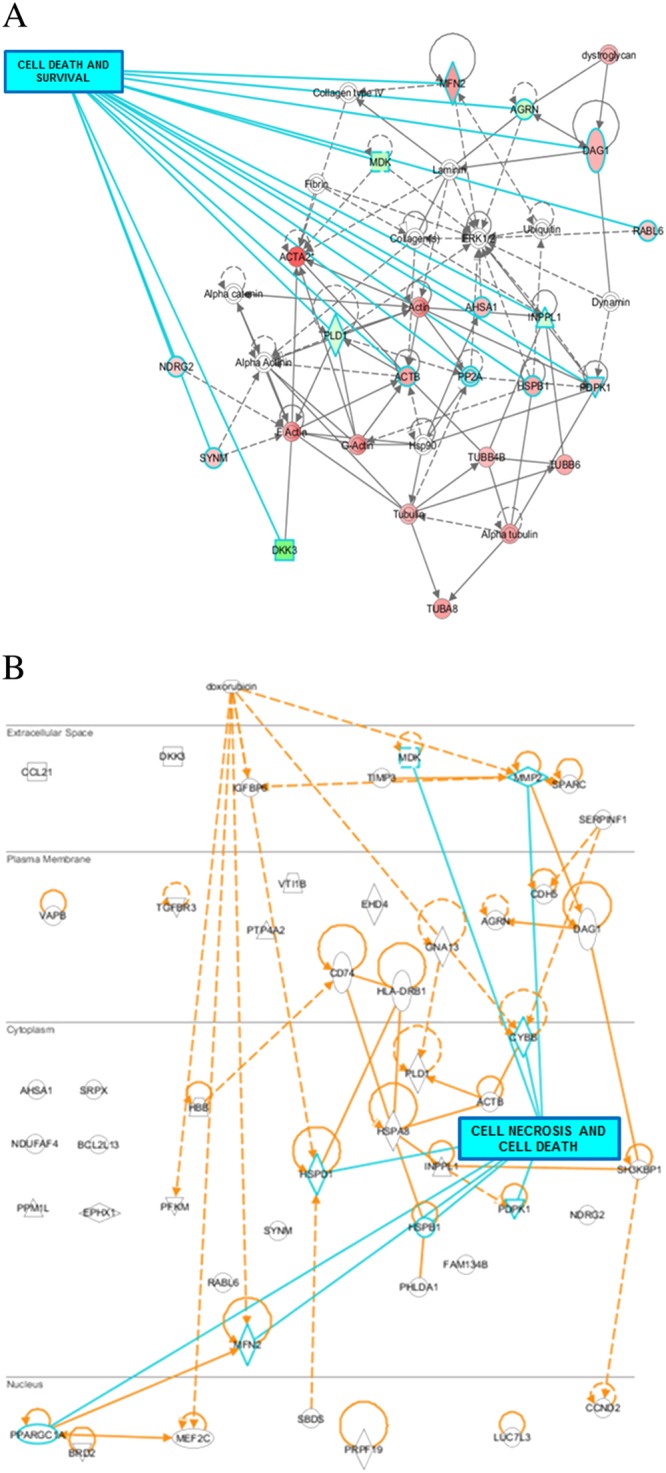
Identification of differentially regulated genes within the cellular assembly and organization, cellular function and maintenance and cell death and survival network. (A) Gene interactions for Network ID 3 in Figure 3 were generated by Ingenuity Pathway Analysis software. Red‐coloured genes are significantly up‐regulated and green‐coloured genes are down‐regulated; the more intense the colour, the higher the level of differential gene expression; uncoloured molecules are not differentially expressed but involved in the network. Solid lines indicate a direct interaction and dashed lines an indirect interaction. The overlay highlights genes specifically involved in cell death and survival mechanisms: ACTB, β‐actin; AGRN aagrin; AHSA1, activator of HSP90 ATPase activity 1; DAG1, dystroglycan 1; DKK3, dickkopf WNT signalling pathway inhibitor 3; HSPB1, heat shock protein binding protein 1; INPPL1, inositol polyphosphate phosphatase like 1; MDK, midkine transcript variant 3; MFN2; NDRG2, N‐myc downstream regulated gene 2; PDPK1; PLD1, phospholipase D1; PP2A, protein phosphatase 2; RABL6, RAB member RAS oncogene family like 6; SYNM, synemin. (B) Cellular location of differentially regulated genes, which interact with DOX (extracellular) and Nox2 (CYBB; at the cytosolic‐plasma membrane junction). Solid lines indicate a direct interaction and dashed lines an indirect interaction. Seven genes specifically involved in cardiomyocyte cell death are highlighted in blue: HSBP1; HSPD1; MDK; MFN2; MMP2; PDPK1; PPARGC1A.

**Figure 5:**
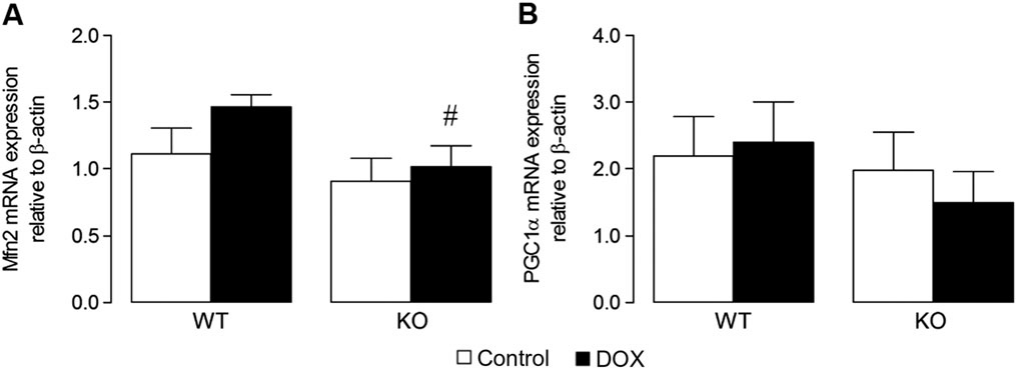
Candidate gene expression in LV tissue from WT and Nox2^−/−^ (KO) DOX‐treated mice. Real‐time RT‐PCR mRNA expression analysis of (A) Mfn2 (*n* = 6) and (B) PGC‐1α (*n* = 9). Data are shown as mean ± SEM and analyses performed using a two‐factor ANOVA followed by Student's unpaired *t*‐test. ^#^ *P* < 0.05 versus WT DOX.

Based on an adjusted *P* value of <0.1 as the selection criterion, 152 mapped genes were found to be differentially expressed in LV tissue from WT versus Nox2^−/−^ DOX‐treated mice, and these are detailed in Supporting Information Table S2: (A) 92 up‐regulated genes and (B) 60 down‐regulated genes. Ingenuity Pathway Analysis highlighted a number of networks, which related to functional sub‐sets. Figure 3 outlines the top 12 networks ranked on the basis of the number of focus molecules from the data set cross referenced against the library of networks. In the context of this investigation, the network ranked third highest is of particular interest – Cellular Assembly and Organization, Cellular Function and Maintenance, and Cell Death and Survival, which included 18 differentially regulated genes involved in these highly complex systems. Shown schematically in Figure 4A with highlighting of 15 genes particularly involved in Cell Death and Survival, it is not surprising to observe convergence on ERK1/ERK2, members of the MAPK super family that control cell fate through proliferation, apoptosis and necrosis pathways. Focusing on particular pathways involved in cardiomyocyte cell death that comprise Nox2 and activation by DOX, Figure 4B shows networks, which include six relevant molecules: midkine and MMP2 in extracellular space; HSP1, Mfn2; 3‐phosphoinositide dependent protein kinase 1 (PDK1) in cytoplasm‐ and PPARγ coactivator‐1α (PGC‐1α) in the nucleus. It should be noted that some of these genes were not necessarily differentially expressed based on high‐stringency single gene analysis (*P* < 0.05), specifically MMP2 and PDK1, but rather represented central nodes within the highlighted cell death signalling pathways (selected by our lower stringency approach, *P* < 0.1; see Methods), which may be of potential significance with regard to the observed effects of DOX. In an Ingenuity Downstream Effects Analysis, just two of the above‐mentioned genes were predicted to increase the function: up‐regulated Mfn2, a mitochondrial membrane protein, and down‐regulated midkine, a heparin‐binding growth factor/cytokine. It is of note, however, that while up‐regulated PGC‐1α was predicted to decrease the cell death function, it is a known activator of cardiac Mfn2 (Li *et al.,* 2009). Indeed, as shown in Figure 4B, both PGC‐1α and Mfn2 are implicated in DOX‐related mechanisms, downstream of Nox2. In validation of the microarray data for Mfn2 and PGC‐1α, showing fold changes in DOX‐treated WT versus Nox2^−/−^ mice of 1.5 and 1.7, respectively, mRNA analysis of LV tissue samples from all four experimental groups showed increased Mfn2 mRNA expression in DOX‐treated WT versus control, which was largely reduced in DOX‐treated Nox2^−/−^ mice with no difference in controls (Figure 5A); although not statistically significant, a similar trend was observed for PCG‐1α mRNA expression (Figure 5B). Based on a robust microarray experiment and analysis, along with reproducibility of DOX‐induced Mfn2 activation in a separate experimental set, and the supporting literature from the Ingenuity Knowledge Base, the potential of up‐regulated Mfn2 to influence cardiomyocyte survival was clearly apparent.

### Investigation of Mfn2 involvement in Nox2‐dependent signalling mechanisms underlying DOX‐induced cardiomyocyte apoptosis

**Figure 6:**
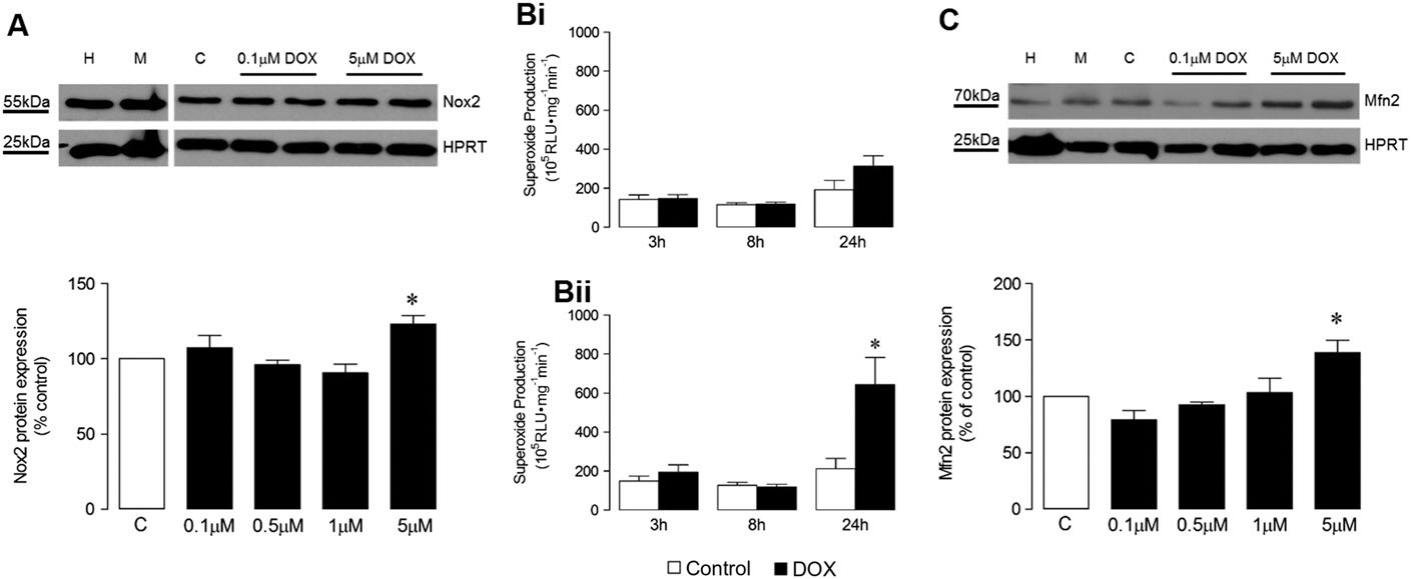
Effect of DOX concentration and time of incubation on superoxide production, gene and protein expression in HL‐1 cardiomyocytes. (A) Representative western blot showing effects of DOX concentration on Nox2 protein expression at 24 h and its quantification (*n* = 5); (B) NADPH oxidase activity in the presence of (i) 0.5 μM and (ii) 5 μM DOX assessed by lucigenin‐enhanced chemiluminescence over 24 h (both *n* = 5); (C) Representative western blot showing effects of DOX concentration on Mfn2 protein expression at 24 h and its quantification (*n* = 5). HPRT, hypoxanthine‐guanine phosphoribosyltransferase endogenous control; H, HeLa cell lysate positive control; M, MCF‐7 cell lysate positive control; C, Control (normal medium). Data are shown as mean ± SEM and analyses performed using one‐factor ANOVA (Kruskal–Wallis test) and Dunn's *post hoc* test (A, C) or two‐factor ANOVA with Student's paired *t*‐test, as indicated (B). *P < 0.05 versus Control.

**Figure 7:**
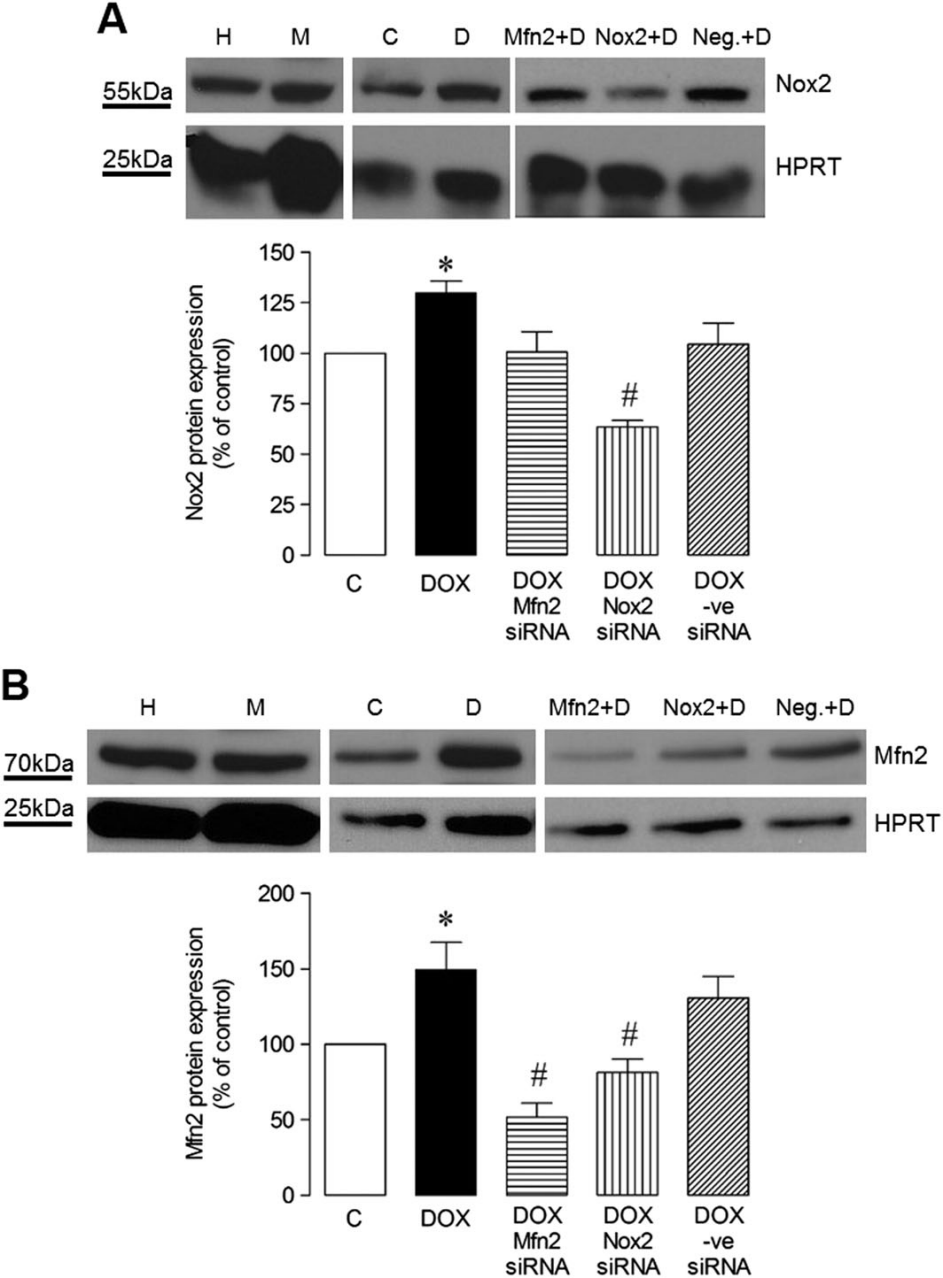
Effect of siRNA gene silencing on DOX‐induced Nox2 and Mfn2 protein expression in HL‐1 cardiomyocytes. Representative western blots of protein expression induced by DOX (5 μM) at 24 h under control conditions and in the presence of Silencer Select® siRNAs (Mfn2, Nox2, Universal negative control, 200 M) and its quantification: (A) Nox2 (*n* = 5) and (B) Mfn2 (*n* = 6). H, HeLa cell lysate positive control; M, MCF‐7 cell lysate positive control; C, Control (normal medium); D, DOX; Mfn2, Mfn2 siRNA; Nox2, Nox2 siRNA; Neg., negative control siRNA. Data are shown as mean values ± SEM and analyses performed using one‐factor ANOVA (Kruskal–Wallis test) and Dunn's *post hoc* test. *P < 0.05 versus Control; ^#^ *P* < 0.05 versus negative control DOX + siRNA.

In order to assess the relevance of HL‐1 cardiomyocytes as a model for study of mechanisms underlying DOX‐induced oxidative stress, the temporal and concentration‐dependent effects of DOX on protein expression/activity of Nox2 and Mfn2 were investigated. DOX at 5 μM increased Nox2 protein at 24 h (Figure A), and this was reflected in increased superoxide production at this concentration (Figure Bii), but not at a lower DOX concentration of 0.5 μM (Figure Bi). Similarly, Mfn2 protein expression was increased by 5 μM DOX at 24 h (Figure 6C), which was subsequently confirmed in a time course experiment in which values were increased 2.1‐ and 2.3‐fold at 24 and 48 h respectively. It was subsequently confirmed that treatment with a DOX concentration of 5 μM for 24 h produced a large increase in caspase 3/7 activity in HL‐1 cardiomyocytes along with increased cell death, consistent with a pattern of apoptosis with secondary necrosis under these experimental conditions (Supporting Information Fig. S5). A further reduction in cell viability at a higher DOX concentration (50 μM) was not matched by an increased cytotoxicity readout. Furthermore, caspase 3/7 activity was reduced, indicating that when subjected to extreme stress, it is possible to miss the apoptotic window in which cells display their characteristic features. For this reason, lower DOX concentrations were used in subsequent investigations of Nox2 and Mfn2 signalling in cell death mechanisms using gene silencing and pharmacological approaches. Initially, it was established that DOX‐stimulated Nox2 protein expression could be abrogated using specific gene silencing, whilst knockdown of Mfn2 did not affect Nox2 protein level by comparison with the non‐targeting negative control siRNA (Figure 7A). In contrast, DOX‐stimulated Mfn2 protein expression was abolished using Mfn2 siRNA, whilst knockdown of Nox2 reduced Mfn2 protein level by ~50% (Figure 7B), indicating possible regulation of Mfn2 by Nox2.

**Figure 8:**
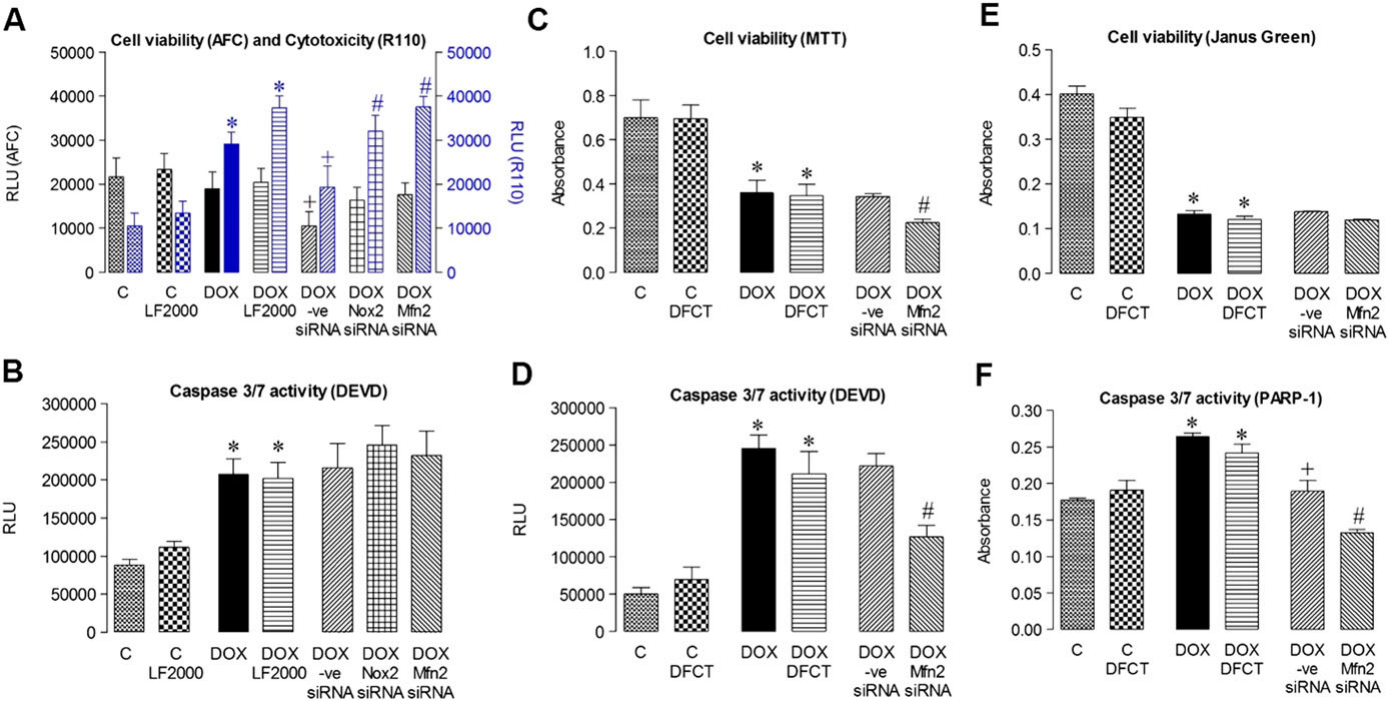
Effect of Mfn2 and Nox2 gene silencing on HL‐1 cardiomyocyte survival. ApoTox‐Glo™ Triplex assay of cells incubated with normal medium (Control, C) or with DOX (5 μM) for 24 h without or with previous transfection using LF2000 alone or with Silencer Select® Universal Negative Control, Mfn2 or Nox2 siRNAs (200 M) for 24 h detected by fluorescence: (A) Cell viability (AFC) and cytotoxicity (R110); and by luminescence: (B) Caspase 3/7 activity (cleavage of DEVD; all *n* = 5, three to four replicates in each experiment, which were averaged to provide a single value): using reverse transfection with Dharmafect (DFCT) and ON‐TARGET plus SMARTpool siRNAs (24 h, 100 nM) and DOX (3 μM, 24 h): (C) Colorimetric readout of cell viability (MTT), (D) Caspase‐Glo 3/7 assay of caspase 3/7 activity (DEVD); (E) Colorimetric readout of cell viability (Janus Green whole cell stain); (F) Colorimetric In‐Cell elisa of cleaved PARP‐1 (all *n* = 7). RLU, relative light units. Data are shown as mean value ± SEM and analyses performed using a one‐factor ANOVA followed by Bonferroni *post hoc* test. *P < 0.05 versus Control or Control + transfection agent; ^+^ *P* < 0.05 DOX ± ve siRNA versus DOX + transfection agent; ^#^ *P* < 0.05 DOX + Nox2 or Mfn2 siRNA versus DOX ± ve siRNA.

Using the ApoTox‐Glo™ Triplex assay, in an ‘add‐mix measure’ format, the simultaneous measurement of cell viability and cytotoxicity produced unusual reductions of both of these measures when Ambion Silencer Select® negative control siRNA was used prior to DOX treatment: by comparison, targeting of Nox2 or Mfn2 was observed to produce increases in cytotoxicity, although not versus the DOX+LF2000 transfection control (Figure 8A). Cell viability as well as cell phenotype of a sample treated with negative control siRNA should remain comparable with that of an untreated sample, that is, the transfection control. Furthermore, live and dead cell measures are normally inversely related, but here, there was a concomitant decrease, not increase, in the cytotoxic response for negative control siRNA versus transfection control. The fact that there was a reduction in both readouts indicates the possibility that colour quenching of the fluorimetric readout (Niles *et al.,* 2007) might have occurred under conditions of negative control siRNA transfection. Alternatively, an added cytotoxic effect by targeting a survival gene could produce a similar profile, as seen in Supporting Information Fig. S5a–b. However, the negative control siRNA appeared to perform well in the caspase 3/7 assay, assessing activity by cleavage of the tetrapeptide sequence DEVD, such that there was no difference by comparison with the transfection control (Figure 8B). Gene‐specific targeting of Nox2 or Mfn2 had no effect on the level of apoptosis induced by DOX under these conditions; it was therefore concluded that the level of knockdown may be insufficient and so an alternative experimental gene silencing strategy was adopted and a lower, likely less, toxic concentration of DOX used. Indeed, in reverse‐transfected HL‐1 cardiomyocytes carried out in suspension using Dharmacon reagents, prior to seeding and DOX treatment (3 μM), Mfn2 knockdown exceeded 90%. Subsequently, using the same caspase 3/7 assay, Mfn2 gene silencing significantly reduced activity versus a non‐targeting siRNA (Figure 8D), although also reducing cell viability, assessed using the MTT colorimetric assay of cell metabolic activity (Figure 8C). Using a further index of caspase 3/7 activity, measurement by elisa of the 85 kDa fragment from cleavage of PARP‐1 at the DEVD site, knockdown of Mfn2 significantly reduced PARP‐1 cleavage (Figure 8F), also having a detrimental effect on cell integrity, measured using the Janus Green whole cell stain (Figure 8E).

**Figure 9:**
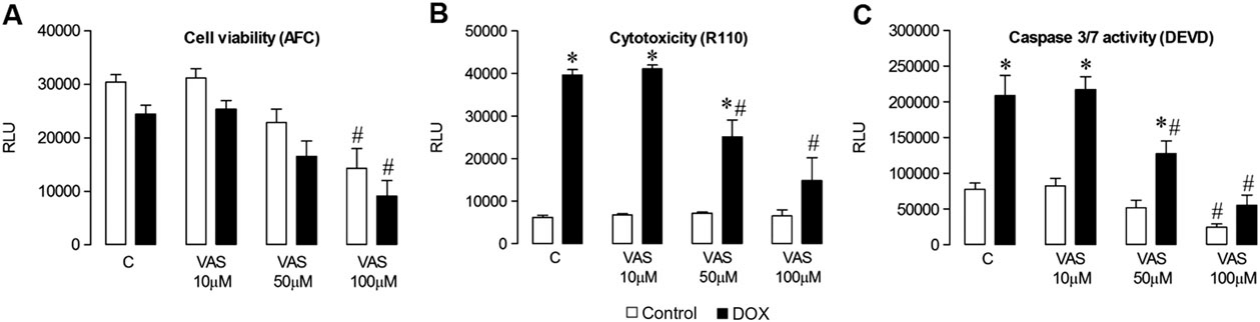
Effect of pharmacological NADPH oxidase inhibition on HL‐1 cardiomyocyte survival. ApoTox‐Glo™ Triplex assay of cells incubated with normal medium (Control, C) or with DOX (5 μM) for 24 h, without or with the pan‐NADPH oxidase inhibitor, VAS2870, at a range of concentrations (VAS 10, 50 and 100 μM). Fluorescent readout of (A) cell viability (AFC) and (B) cytotoxicity (R110). (C) Caspase‐Glo 3/7 assay of caspase 3/7 activity (DEVD). Data are shown as mean value ± SEM (*n* = 6 experiments, three to four replicates in each, which were averaged to provide a single value) and analyses performed using a two‐factor ANOVA followed by paired Student's *t*‐test or Dunnett's test. *P < 0.05 versus respective no DOX; ^#^ *P* < 0.05 versus respective no VAS (Control). RLU, relative light units.

Finally, the effect of pharmacological pan‐NADPH oxidase inhibition on DOX‐induced cardiotoxicity was studied in HL‐1 cardiomyocytes using the triazolo pyrimidine, VAS2870, in the ApoTox‐Glo™ assay. When used alone at the highest concentration (100 μM), VAS2870 produced a significant reduction in cell viability, despite showing no cytotoxic effects or increases on apoptosis, as determined by caspase 3/7 activity. However, when used at a concentration of 50 μM, which had no basal effects on the cells, VAS2870 markedly attenuated DOX‐induced increases in both cytotoxicity and apoptosis, suggesting that these processes occur at least partly due to activation of NADPH oxidases.

## Discussion

Despite strong evidence supporting a role for Nox2‐derived ROS in DOX‐induced cardiotoxicity and their known involvement in established remodelling pathways, the precise mechanisms underlying Nox2 activation in this setting remain unknown. We sought, therefore, to identify and examine potential new mechanisms underlying NADPH oxidase‐dependent downstream signalling in response to DOX treatment in cardiomyocytes.

As a basis for mechanistic studies, the initial objective was to characterize a murine model of DOX‐induced cardiotoxicity in which Nox2‐specific effects could be examined, and this was achieved using WT and Nox2^−/−^ mice as in our previous study (Zhao *et al.,* 2010), except that sampling was performed at 4 weeks rather than 8 weeks after initial treatment. Consistent with previous findings, genetic deletion of Nox2 protected mice against DOX‐induced (i) development of cardiac contractile dysfunction, specifically normalizing systolic and diastolic function; (ii) cardiomyocyte atrophy, attenuating reductions in both LV mass and cardiomyocyte cross‐sectional area; (iii) cardiomyocyte apoptosis, diminishing an increase of TUNEL‐positive cells and of caspase 3/7 activity; and (iv) superoxide generation, reducing increased levels observed in WT mice. It was also established that DOX‐induced ROS production in this setting was Nox2‐derived, not due to modulation of endogenous antioxidant capacity or nitrosative stress, but consistent with DOX‐stimulated expression of Nox2. Taken together, these current data consolidate convincing previous evidence that DOX‐induced Nox2 NADPH oxidase‐derived ROS are involved in progression towards cardiomyocyte apoptosis. As such, the experimental model of DOX‐induced cardiotoxicity characterized here, examined 4 weeks post‐treatment, appears suitable for investigation of novel genes and signalling pathways regulated by Nox2.

In subsequent microarray analysis comparing hearts from DOX‐treated WT and Nox2^−/−^ mice, cell death and survival functions were found to contribute the most relevant and significant network to this dataset, in accordance with many previous findings relating to transcriptional analysis of cardiac DOX effects, identifying the importance of the protein ubiquitination response amongst others (Deng *et al.,* 2007; Thandavarayan *et al.,* 2010; Tokarska‐Schlattner *et al.,* 2010; Zhang *et al.,* 2012; Sishi *et al.,* 2013). In this study, we chose to specifically focus on the role of Nox2 as a novel aspect of DOX‐mediated signalling in this setting. Probing the identified network for Nox2 and its interrelationships with pathways linked to cardiac cell fate highlighted a number of relevant genes, some of which would be expected to promote survival or contribute to cell death processes. For example, midkine exerts an acute cytoprotective effect on ischaemia–reperfusion injury, at least in part due to its anti‐apoptotic effect (Kadomatsu *et al.,* 2014); however, in DOX‐treated WT hearts, the expression of midkine was down‐regulated by comparison with Nox2^−/−^ hearts, so the involvement of this growth factor in DOX‐Nox2 apoptotic signalling may be questionable, although in a chronic treatment regime, it is possible that midkine may underlie an opposite adaptive mechanism. Similarly, down‐regulation of MMP2 may be a late phase adaptation, since dysregulation of myocardial MMPs is generally regarded as an early contributory mechanism towards initiation and progression of heart failure. In particular, enhancement of MMP2 in cardiomyocytes in response to DOX has been identified as redox‐dependent (Spallarossa *et al.,* 2006; Mukhopadhyay *et al.,* 2009; Bartekova *et al.,* 2015). Other significant changes induced by DOX and mediated by Nox2 indicated up‐regulation of gene expression and included increased phosphoinositide‐dependent protein kinase‐1, which is an important mediator of PI3K signalling, promoting cardiomyocyte survival via the PI3K‐Akt pathway (An *et al.,* 2013; Kitamura *et al.,* 2014). Similarly, DOX‐induced up‐regulation of HSP binding protein 1, which is implicated in maintaining redox homeostasis by upholding glutathione levels (Christians *et al.,* 2012), may signify an attempt to promote survival by counteracting increased ROS production. Consistent with this argument, the up‐regulation of heat shock factor binding protein 1 by DOX, by repressing the transcriptional activity of heat shock factor‐1, which can potentiate apoptosis through increased HSP25 (Vedam *et al.,* 2010), might be expected to represent a counter‐regulatory mechanism for cell survival.

The most exceptional finding from our gene array analysis was the identification of DOX‐Nox2‐mediated up‐regulation of Mfn2, a protein found in the outer mitochondrial membrane, which plays key roles in determining mitochondrial morphology and regulation of the fusion process. Changes in mitochondrial morphology have been linked to apoptotic cell death (Ong and Hausenloy, 2010), and Mfn2, independent of its pro‐fusion properties, can bind with a pro‐apoptotic member of the Bcl‐2 family, Bax (Wang *et al.,* 2010). Mfn2 is able to form a functional unit with the mitochondrial fission protein, dynamin‐related protein 1, and Bax at the outer mitochondrial membrane to mediate apoptotic cell death (Karbowski *et al.,* 2002). In agreement, a considerable number of studies identified in the Ingenuity Knowledge Base were found to support the idea that increased Mfn2 in cardiomyocytes predicted an adverse outcome, promoting cell death through apoptotic mechanisms. For example, Shen *et al.* (2007) demonstrated that Mfn2 mediates oxidative stress‐induced apoptotic cell death in neonatal cardiomyocytes. Additionally, siRNA inhibition of Mfn2 prevented oxidative stress‐induced apoptotic cell death in H9c2 cardiomyocytes (Karbowski *et al.,* 2002). Strikingly, Mfn2 is reported to protect the heart against ischaemia–reperfusion injury and ROS‐mediated damage (Papanicolaou *et al.,* 2011). It was therefore concluded that Mfn2 not only serves to maintain mitochondrial morphology in cardiomyocytes but also promotes mitochondrial permeability transition activation in response to Ca^2+^ stimulation or ROS generation, predisposing the cells to a number of cell‐death‐inducing stimuli. In substantiating our choice of Mfn2 as a primary candidate underlying DOX‐stimulated Nox2 signalling, it was noted that PGC‐1α, which was also identified from the array analysis as a potential gene involved in Nox2‐dependent cardiomyocyte apoptosis, is reported to up‐regulate Mfn2 expression in response to metabolic demand (Soriano *et al.,* 2006; Romanello and Sandri, 2013).

Further, to the identification of cardiomyocyte apoptosis as a major element in the DOX‐induced cardiotoxic response and discovery that up‐regulation of Mfn2 was strongly linked, the final part of this investigation examined the functional relevance of Mfn2 and its relationship to Nox2 in cell death processes using gene silencing in HL‐1 cardiomyocytes. Important characteristics of the mouse model used in mRNA profiling of cardiac DOX‐Nox2 signalling were replicated in HL‐1 cardiomyocytes treated with DOX (5 μM), namely increased superoxide production, and up‐regulation of Nox2 and Mfn2 protein expression. Furthermore, the cell death profile induced by DOX in this setting is consistent with increased apoptosis leading to secondary necrosis, whilst the concentrations of DOX used in this study (0.5–5 μM) are relevant to plasma levels found clinically up to 1 h post‐treatment (0.1–10 μM) (Anderson *et al.,* 1999). In a pharmacological approach, the observed effects of the pan‐NADPH oxidase inhibitor, VAS2870, on DOX (5 μM)‐induced cell death add weight to the involvement of Nox2 signalling in this model, although it should be noted that VAS2870 can exert Nox2‐independent actions (Gatto *et al.,* 2013) and is cytotoxic at high concentrations (Zielonka *et al.,* 2014), which we observed at 100 μM. However, at a concentration of 50 μM, which maintained cell viability, VAS2870 was shown to reduce both cytotoxicity and caspase 3/7 activity.

Again using a DOX concentration of 5 μM and applying specific gene silencing, ~50% knockdown of the Nox2 and Mfn2 proteins was achieved in HL‐1 cardiomyocytes and this was increased to >90% by adopting a reverse transfection protocol. Similar studies have reported the effective use of siRNAs to knockdown both Nox and Mfn2 and inhibit ROS production and apoptosis in cardiac cells, including HL‐1 cardiomyocytes (Yeh *et al.,* 2011), H9c2 cardiomyocytes, (Shen *et al.,* 2007), cultured neonatal rat cardiomyocytes (Papanicolaou *et al.,* 2011) and mouse ventricular cells (Moe *et al.,* 2011). However, while this investigation found no changes in apoptosis in DOX‐treated HL‐1 cardiomyocytes in the presence of Nox2 or Mfn2 knockdown, it is possible that efficacy of transfection may have influenced the measured endpoint. Indeed, highly oxidative tissues such as the heart require constant energy production, and as mitochondria are the powerhouse of the cardiomyocyte comprising a large proportion of cytoplasmic volume, it is possible that there is such a high level of Mfn2 expression in HL‐1 cardiomyocytes that the observed effects of Mfn2 siRNA on cell apoptosis may have underestimated the involvement of Mfn2 (Bach *et al.,* 2003; Papanicolaou *et al.,* 2011). Indeed, when a higher level of knockdown was achieved, a lack of Mfn2 corresponded with reduction of caspase 3/7 activity, assessed by both DEVD and PARP‐1 cleavage. This must be countered, however, by recognition that there was no evidence of reduced cytotoxicity, assessed by the dead cell protease assay (R110). In fact, there was tendency towards reduced cell viability in all three measures, which included two of membrane integrity (live cell protease assay – AFC, Janus Green Whole Cell) and also mitochondrial activity (MTT). It appears, therefore, that in an environment associated with extreme loss of Mfn2 activity, HL‐1 cardiomyocytes are more likely to undergo necrotic transformation; it is also possible that impairment of autophagic processes will impact on mitochondrial quality control (Andres *et al.,* 2015). As further information emerges, it seems that there is equivalent evidence that maintained levels of Mfn2 may be required to counteract cell oxidative stress; for example, further to ROS induced by hypoxia/reoxygenation in cardiomyocytes, up‐regulated Mfn2 expression prevented imbalance in mitochondrial dynamics (Dong *et al.,* 2016). Loss of Mfn2 also delayed membrane depolarization in isolated cardiomyocytes from adult Mfn2^−/−^ mice, leading to the suggestion that Mfn2 may function to control mitochondrial permeability transition pore opening (Papanicolaou *et al.,* 2011). Similarly, cardiac‐specific deletion of Mfn2 produced dissipation of mitochondrial membrane potential and elevated ROS production (Chen *et al.,* 2012), whilst overexpression of Mfn2 was found to increase the percentage of cells containing elongated mitochondria, thereby reducing mitochondrial permeability transition pore opening and cell death after simulated ischaemia/reperfusion injury (Ong *et al.,* 2010). It also appears that Mfn2 serves an essential role in maintaining mitochondrial coenzyme Q levels in mouse hearts, thereby promoting optimal function of the respiratory chain (Mourier *et al.,* 2015).

In summary, therefore, it is probably true to say that the participation of Mfn2 in control of cardiomyocyte life or death is complex and depends upon its level of expression (Schrepfer and Scorrano, 2016). Nonetheless, the results of this study clearly demonstrate that while DOX through NADPH oxidase signalling in general can have a detrimental effect on cardiomyocyte survival, a particular Nox2‐stimulated pathway including Mfn2 may signify an attempt to maintain mitochondrial biogenesis.

In consideration of this novel premise, it must be taken into account that our investigation has limitations primarily that the mouse model may not truly reflect salient features of DOX‐induced cardiotoxicity in humans, because of differences in drug metabolism and/or cardiac structure and function, and sensitivity to cardiac injury, all of which may be influenced by ageing and co‐morbidities (Madonna *et al.,* 2015). Preferentially, studies in human cardiac tissue would be performed, but being largely unobtainable, there has been increasing interest in disease modelling using human embryonic stem cell and induced pluripotent stem cell‐derived cardiomyocytes (Madonna *et al.,* 2016; Maillet *et al.,* 2016), although this also is not without criticism. Another approach is to examine peripheral blood mononuclear cells (PBMCs), in which the transcriptome in DOX‐exposed PBMCs is highly similar to that in treated cardiomyocytes (Todorova *et al.,* 2012). Of particular relevance to the findings of the current study, it would be of interest to extend investigation of the role of Mfn2 after DOX treatment using patient‐derived PBMCs, since systemic mitochondrial pathologies have been shown to correlate in PBMCs and in cardiac tissues (Lipshultz *et al.,* 2016).

The potential for activation of Mfn‐2 as a therapeutic strategy for cardioprotection in ischaemic disease and heart failure has recently received considerable attention (Ong *et al.,* 2015; Walters *et al.,* 2016). This has been intensified by recognition that Mfn2 may play a critical role in cell‐based therapies promoting the differentiation of stem cells into cardiomyocytes (Kasahara *et al.,* 2013; Suliman *et al.,* 2016). Indeed, DOX‐induced cardiomyopathy is associated with depletion and senescence of the cardiac progenitor cell pool in both rat and human hearts, permanently impairing their function (de Angelis *et al.,* 2010; Piegari *et al.,* 2013). Therefore, a pharmacological strategy involving Mfn2 that could potentially prevent degeneration of both adult cardiac cells and the resident stem cell pool seems an attractive idea. Such an approach may be enabled by the identification of a small natural molecule, 15‐oxospiramilactone, which, through inhibition of a mitochondria‐localized deubiquitinase, increases Mfn2 activity (Yue *et al.,* 2014), although effects have yet to be demonstrated in relevant models that could indicate potential targeting of this mechanism for translation to the clinic.

## Author contributions

D.McL, Y.Z, K.M.O′N, D.J.G and B.J.McD conceived and designed the experiments; D.McL, K.M.O′N, Y.Z., K.S.E and A.M.K performed the experiments; D.McL, Y.Z, K.M.O′N, K.S.E, P.D.D, D.J.G and B.J.McD analysed the data and drafted relevant text; D.McL, D.J.G and B.J.McD wrote the manuscript.

## Conflict of interest

The authors declare no conflicts of interest.

## Declaration of transparency and scientific rigour

This Declaration acknowledges that this paper adheres to the principles for transparent reporting and scientific rigour of preclinical research recommended by funding agencies, publishers and other organisations engaged with supporting research.

## Supporting information


**Table S1** Real‐time RT‐PCR primer sequences.
**Table S2** Differentially expressed genes in LV tissue from WT vs. Nox−/− mice.
**Figure S1** Time course of contractile dysfunction in response to DOX treatment.
**Figure S2** Effect of DOX on LV NOS isoenzyme and antioxidant gene mRNA expression.
**Figure S3** Quality control assessment of Illumina MouseWG‐6 v2.0 microarray.
**Figure S4** Comparison of normalized transcript data within samples from WT and Nox2−/− mice.
**Figure S5** Concentration responses for optimization of experimental conditions for the ApoTox‐Glo Triplex assay in HL‐1 cardiomyocytes.
