# Supplementary material for: Signalling mechanisms underlying doxorubicin and Nox2 NADPH oxidase‐induced cardiomyopathy: involvement of mitofusin‐2
Source: Br J Pharmacol. 2017 Apr 22;174(21):3677–95. doi: 10.1111/bph.13773 (PMC5647180; doi:10.1111/bph.13773)
Supplement: Supplementary file 1 — Table S1 Real‐time RT‐PCR primer sequences. Table S2 Differentially expressed genes in LV tissue from WT vs. Nox−/− mice. Figure S1 Time course of contractile dysfunction in response to DOX treatment. Figure S2 Effect of DOX on LV NOS isoenzyme and antioxidant gene mRNA expression. Figure S3 Quality control assessment of Illumina MouseWG‐6 v2.0 microarray. Figure S4 Comparison of normalized transcript data within samples from WT and Nox2−/− mice. Figure S5 Concentration responses for optimization of experimental conditions for the ApoTox‐Glo Triplex assay in HL‐1 cardiomyocytes. [file BPH-174-3677-s001.pdf]

SUPPORTING INFORMATION

SIGNALLING MECHANISMS UNDERLYING DOXORUBICIN AND NOX2 NADPH OXIDASE-INDUCED CARDIOMYOPATHY: INVOLVEMENT OF MITOFUSIN-2

Declan McLaughlin[1], Youyou Zhao[1], Karla M. O'Neill[1], Kevin S. Edgar[1], Philip D. Dunne[2], Anna M. Kearney[1], David J. Grieve[1], Barbara J. McDermott[1]

Queen's University Belfast, [1]Wellcome-Wolfson Institute for Experimental Medicine, and [2]Centre for Cancer Research & Cell Biology, Belfast BT9 7AE, UK

TABLE S1: Real-time RT-PCR primer sequences

Gene	Forward Sequence	Reverse Sequence
**β-actin**	5’ CGT GAA AAG ATG ACC CAG ATC A 3’	5’ TGG TAC GAC CAG AGG CAT ACA G 3’
**Nox2**	5’ ACT CCT TGG GTC AGC ACT GG 3’	5’ GTT CCT GTC CAG TTG TCT TCG 3’
**GPx1**	5’ AGG CTC ACC CGC TCT TTA CC 3’	5’ GGG TCG TCA CTG GGT GTT G 3’
**NOS1 (nNOS)**	5’ GAC TGA TGG CAA GCA TGA CTT C 3’	5’ GCC CAA GGT AGA GCC ATC TG 3’
**NOS2 (iNOS)**	5’ TGA CGG CAA ACA TGA CTT CAG 3’	5’ GCC ATC GGG CAT CTG GTA 3’
**NOS3 (eNOS)**	5’ TCT GCG GCG ATG TCA CTA TG 3’	5’ CCA TGC CGC CCT CTG TT 3’
**SOD1**	5’ AGC ATT CCA TCA TTG GCC GTA 3’	5’ TTT CCA CCT TTG CCC AAG TCA 3’
**SOD2**	5’ ACA GAT TGC TGC CTG CTC TAA TCA 3’	5’ TAA GCG TGC TCC CAC ACG TC 3’
**Glutaredoxin1**	5’ CCC TTC CCA CTC CTG CAT T 3’	5’ GGA GGT TGA GGC TGA GAA CAC T 3’
**Glutaredoxin2**	5’ TTT GTC AAT GGA CGA TTT ATT GGA 3’	5’GCA GCA ATT TCC CTT CTT TGT G 3’
**Catalase**	5’ TTC AGA AGA AAG CGG TCA AGA AT 3’	5’ GAT GCG GGC CCC ATA GTC 3’
**Peroxisome proliferator-activated receptor gamma coactivator 1-alpha (PGC-1α)**	5’ CAG CCC AGA GTC ACC AAA TGA 3’	5’ TTC CAG AGA GTT CCA CAC TTA AGG T 3’
**Mitofusin 2 (Mfn2)**	5’ CAG TTG GTG TCT GGC ATT GTG 3’	5’ AGG GCC TCA GTG GCA AGA A 3’

Real-time RT-PCR primer sequences were generated using Primer Express Software (Applied Biosystems, UK) and primers obtained from Invitrogen (UK).

TABLE S2: Differentially expressed genes in LV tissue from WT vs. $Nox^{-/-}$ mice

(A) Upregulated genes

DEFINITION	Gene ID	logFC	Ave Expr	p value	Adj. p value
integrin beta 1 binding protein 3 (Itgb1bp3)	ITGB1BP3	1.169	7.96	7.31E-04	0.093
hemoglobin, beta adult major chain (Hbb-b1)	HBB-B1	0.973	9.32	1.77E-04	0.049
actin, alpha 2, smoothcle, aorta (Acta2)	ACTA2	0.783	9.56	3.57E-08	0.001
pleckstrin homology-like domain, family A, member 1 (Phlda1)	PHLDA1	0.695	9.26	3.36E-05	0.028
DEAD (Asp-Glu-Ala-Asp) box polypeptide 24 (Ddx24)	DDX24	0.627	8.57	8.66E-06	0.016
heterogeneous nuclear ribonucleoprotein A2/B1 (Hnrnpa2b1), transcript variant 2	HNRNPA2B1	0.615	9.54	4.05E-05	0.028
RIKEN cDNA 1810015C04 gene (1810015C04Rik), transcript variant 2	1810015C04RIK	0.588	11.31	2.73E-04	0.063
PREDICTED: similar to mitofusin 2, transcript variant 1 (LOC100044767)	MFN2	0.573	8.06	1.73E-04	0.048
phosphofructokinase,cle (Pfkm)	PFKM	0.562	8.82	3.26E-04	0.068
tubulin, alpha 8 (Tuba8)	TUBA8	0.548	9.49	9.23E-05	0.039
SH3-domain kinase binding protein 1 (Sh3kbp1)	SH3KBP1	0.543	9.96	2.37E-06	0.010
	HRMT1L2	0.518	9.31	8.79E-04	0.096
heat shock protein 1 (chaperonin) (Hspd1)	HSPD1	0.516	9.87	5.78E-06	0.012
cyclin D2 (Ccnd2)	CCND2	0.512	9.60	1.88E-05	0.024
actin, beta, cytoplasmic (Actb)	ACTB	0.509	8.58	9.59E-04	0.099
PREDICTED: similar to human protein homologous to DROER protein (LOC100042777)	LOC100042777	0.503	8.89	5.49E-05	0.034
peroxisome proliferative activated receptor, gamma, coactivator 1 alpha (Ppargc1a)	PPARGC1A	0.500	8.92	3.58E-06	0.010
trafficking protein particle complex 3 (Trappc3)	TRAPPC3	0.500	8.34	4.08E-05	0.028
Nipped-B homolog (Drosophila) (Nipbl), transcript variant A	NIPBL	0.489	8.58	1.73E-05	0.024
tubulin, beta 6 (Tubb6)	TUBB6	0.487	8.62	3.93E-04	0.076
transmembrane protein 63b (Tmem63b)	TMEM63B	0.473	9.59	4.19E-05	0.028
heat shock protein 1 (Hspb1)	HSPB1	0.471	9.84	1.97E-05	0.024
acidic (leucine-rich) nuclear phosphoprotein 32 family, member E (Anp32e)	ANP32E	0.468	9.10	1.12E-04	0.039
calcium channel, voltage-dependent, alpha2/delta subunit 1 (Cacna2d1)	CACNA2D1	0.467	8.77	2.92E-05	0.028
dystroglycan 1 (Dag1)	DAG1	0.438	9.46	4.40E-04	0.078
peroxisomal biogenesis factor 11a (Pex11a)	PEX11A	0.433	8.13	3.29E-05	0.028
enhancer of rudimentary homolog (Drosophila) (Erh)	ERH	0.430	9.14	1.49E-04	0.045
RIKEN cDNA 3300001P08 gene (3300001P08Rik)	3300001P08RIK	0.423	9.93	3.48E-04	0.070
heat shock protein 8 (Hspa8)	HSPA8	0.410	10.63	6.99E-05	0.036
vesicle transport through interaction with t-SNAREs 1B homolog (Vti1b)	VTI1B	0.406	8.02	6.13E-04	0.087

	D630048P19RIK	0.396	8.60	2.67E-05	0.028
tubulin, beta 2c (Tubb2c)	TUBB2C	0.394	11.44	2.81E-04	0.063
methionine adenosyltransferase II, alpha (Mat2a)	MAT2A	0.388	8.11	1.42E-04	0.045
AHA1, activator of heat shock protein ATPase homolog 1 (yeast) (Ahsa1)	AHSA1	0.387	8.14	4.42E-04	0.078
protein tyrosine phosphatase 4a2 (Ptp4a2)	PTP4A2	0.384	8.12	1.87E-04	0.050
transportin 3 (Tnpo3)	TNPO3	0.372	8.32	2.79E-04	0.063
sushi-repeat-containing protein (Srpx)	SRPX	0.371	7.08	6.37E-04	0.087
forkhead box N3 (Foxn3)	FOXN3	0.370	8.81	3.19E-04	0.067
	3000003G13RIK	0.369	9.18	2.15E-04	0.055
solute carrier family 25 (mitochondrial carnitine/acylcarnitine translocase), member 20 (Slc25a20)	SLC25A20	0.368	8.85	1.00E-04	0.039
PREDICTED: predicted gene, EG433144 (EG433144)	EG433144	0.364	9.15	2.61E-04	0.061
guanine nucleotide binding protein (G protein), beta 5 (Gnb5), transcript variant 1	GNB5	0.354	8.13	1.24E-04	0.040
RIKEN cDNA 2410003P15 gene (2410003P15Rik)	2410003P15RIK	0.353	9.32	1.53E-04	0.045
aminolevulinic acid synthase 1 (Alas1)	ALAS1	0.352	12.39	3.96E-05	0.028
LIM domain binding 3 (Ldb3), transcript variant 6	LDB3	0.351	10.09	6.45E-05	0.036
peroxisome proliferative activated receptor, gamma, coactivator 1 beta (Ppargc1b)	PPARGC1B	0.351	9.58	7.38E-04	0.093
leucine-rich repeats and transmembrane domains 1 (Lrtm1)	LRTM1	0.349	9.86	6.77E-05	0.036
G kinase anchoring protein 1 (Gkap1)	GKAP1	0.347	8.57	4.48E-04	0.079
TNNI3 interacting kinase (Tnni3k)	TNNI3K	0.344	8.26	7.64E-05	0.036
high density lipoprotein (HDL) binding protein (Hdlbp)	HDLBP	0.339	8.37	2.57E-04	0.061
heterogeneous nuclear ribonucleoprotein H1 (Hnrph1)	HNRPH1	0.338	9.43	6.20E-04	0.087
desmuslin (Dmn), transcript variant 3	DMN	0.336	7.36	4.23E-04	0.078
3-phosphoinositide dependent protein kinase-1 (Pdpk1), transcript variant 2	PDPK1	0.334	7.99	3.40E-04	0.070
BCL2-like 13 (apoptosis facilitator) (Bcl2l13), nuclear gene encoding mitochondrial protein	BCL2L13	0.331	8.21	5.08E-04	0.081
RIKEN cDNA 2810474O19 gene (2810474O19Rik)	2810474O19RIK	0.327	7.44	7.54E-05	0.036
proline synthetase co-transcribed (Prosc), transcript variant 3	PROSC	0.319	8.19	2.57E-04	0.061
regulator of calcineurin 2 (Rcan2), transcript variant 1	RCAN2	0.318	9.53	1.15E-04	0.039
RIKEN cDNA B230208H17 gene (B230208H17Rik) XM_897418 XM_897429 XM_897439 XM_897452 XM_897459 XM_914394 XM_923230 XM_923233 XM_923237 XM_923239 XM_923241 XM_923243 XM_923244 XM_923249 XM_923252	B230208H17RIK	0.306	7.85	5.54E-04	0.085
SNRPN upstream reading frame (Snurf)	SNURF	0.306	10.46	8.73E-04	0.096
	GNA13	0.306	8.17	8.88E-04	0.096
SMT3 suppressor of mif two 3 homolog 3 (yeast) (Sumo3)	SUMO3	0.304	8.33	4.79E-04	0.081
insulin induced gene 2 (Insig2)	INSIG2	0.303	9.52	1.70E-04	0.048
zinc finger CCCH type containing 13 (Zc3h13)	ZC3H13	0.303	7.13	2.31E-04	0.057

	LOC331507	0.302	9.47	1.49E-04	0.045
RIKEN cDNA 1700113I22 gene (1700113I22Rik)	1700113I22RIK	0.301	9.25	1.21E-04	0.040
splicing factor, arginine/serine-rich 10 (transformer 2 homolog, Drosophila) (Sfrs10)	SFRS10	0.299	8.01	9.80E-04	0.099
EH-domain containing 4 (Ehd4)	EHD4	0.293	12.64	8.95E-04	0.096
Cd63 antigen (Cd63), transcript variant 2	CD63	0.289	8.70	5.64E-04	0.085
	LOC384888	0.288	7.60	8.30E-04	0.096
RIKEN cDNA 1110007M04 gene (1110007M04Rik)	1110007M04RIK	0.288	11.27	4.42E-04	0.078
PRP19/PSO4 pre-mRNA processing factor 19 homolog (S cerevisiae) (Prpf19)	PRPF19	0.288	8.83	7.08E-04	0.092
myocyte enhancer factor 2C (Mef2c)	MEF2C	0.287	9.39	9.84E-04	0.099
calumenin (Calu), transcript variant 2	CALU	0.286	7.45	8.81E-04	0.096
dynein light chain LC8-type 2 (Dynll2)	DYNLL2	0.285	7.25	4.04E-04	0.076
adipose differentiation related protein (Adfp)	ADFP	0.283	7.71	6.00E-04	0.087
transmembrane protein 49 (Tmem49)	TMEM49	0.278	8.53	9.87E-04	0.099
PREDICTED: similar to medium-chain acyl-CoA dehydrogenase (LOC333331), misc RNA	LOC333331	0.277	12.15	8.97E-04	0.096
PREDICTED: similar to mitochondrial ribosomal protein S5 (LOC667609), misc RNA	LOC667609	0.269	9.11	6.06E-04	0.087
N-myc downstream regulated gene 2 (Ndrg2)	NDRG2	0.268	9.41	9.85E-04	0.099
myeloid leukemia factor 2 (Mlf2)	MLF2	0.263	8.77	7.55E-04	0.095
zinc finger protein 91 (Zfp91)	ZFP91	0.258	7.71	4.37E-04	0.078
Shwachman-Bodian-Diamond syndrome homolog (human) (Sbds)	SBDS	0.256	9.67	3.43E-04	0.070
	C730026J16	0.256	9.02	4.14E-04	0.077
H2A histone family, member Y (H2afy)	H2AFY	0.251	8.31	5.59E-04	0.085
	SIAT7F	0.251	7.10	3.02E-04	0.065
protein phosphatase 1 (formerly 2C)-like (Ppm1l)	PPM1L	0.246	7.73	4.91E-04	0.081
T-box 20 (Tbx20), transcript variant 1	TBX20	0.245	7.27	7.90E-04	0.096
heat shock protein 3 (Hspb3)	HSPB3	0.237	8.78	4.97E-04	0.081
	ZFP445	0.235	7.28	4.77E-04	0.081
adenylosuccinate synthetase like 1 (Adssl1)	ADSSL1	0.229	7.84	9.43E-04	0.098
	MTF2	0.225	8.15	7.26E-04	0.093
tetraspanin 3 (Tspan3)	TSPAN3	0.215	11.15	8.90E-04	0.096

(B) Downregulated genes

DEFINITION	Gene ID	logFC	Ave Expr	p value	Adj. p value
D site albumin promoter binding protein (Dbp)	DBP	-1.203	10.84	7.01E-05	0.036
RIKEN cDNA 1500015O10 gene (1500015O10Rik)	1500015O10RIK	-0.853	7.45	1.57E-05	0.024
Indolethylamine N-methyltransferase (Inmt)	INMT	-0.792	8.87	4.04E-04	0.076
PREDICTED: similar to beta chemokine Exodus-2 (LOC100041504)	LOC100041504	-0.746	8.77	8.91E-04	0.096
Gene model 129, (NCBI) (Gm129)	GM129	-0.721	7.32	5.46E-04	0.084
Chemokine (C-C motif) ligand 21b (Ccl21b)	CCL21B	-0.715	9.60	1.05E-04	0.039
Dickkopf homolog 3 (Xenopus laevis) (Dkk3)	DKK3	-0.695	7.47	6.56E-04	0.088
Vesicle-associated membrane protein, associated protein B and C (Vapb)	VAPB	-0.663	8.22	3.57E-06	0.010
Tissue inhibitor of metalloproteinase 3 (Timp3)	TIMP3	-0.554	10.42	1.13E-04	0.039
Angiopoietin-like 7 (Angptl7)	ANGPTL7	-0.536	7.73	5.86E-05	0.035
Epoxide hydrolase 1, microsomal (Ephx1)	EPHX1	-0.466	9.59	5.59E-06	0.012
Period homolog 2 (Drosophila) (Per2)	PER2	-0.462	8.34	2.12E-04	0.055
Transmembrane protein 82 (Tmem82)	TMEM82	-0.461	7.94	3.03E-04	0.065
Histocompatibility 2, class II antigen E beta (H2-Eb1)	H2-EB1	-0.441	9.32	1.84E-04	0.050
cDNA sequence X99384 (X99384)	X99384	-0.428	8.33	4.18E-05	0.028
Histocompatibility 2, class II antigen A, beta 1 (H2-Ab1)	H2-AB1	-0.420	9.68	2.76E-05	0.028
Mid1 interacting protein 1 (gastrulation specific G12-like (zebrafish)) (Mid1ip1)	MID1IP1	-0.408	10.00	5.10E-04	0.081
Serine (or cysteine) peptidase inhibitor, clade F, member 1 (Serpinf1)	SERPINF1	-0.402	10.39	7.87E-05	0.036
Parvalbumin (Pvalb)	PVALB	-0.389	7.21	9.25E-06	0.016
CD74 antigen (invariant polypeptide of major histocompatibility complex, class II antigen-associated) (Cd74)	CD74	-0.384	9.69	7.90E-04	0.096
Scavenger receptor class A, member 3 (Scara3)	SCARA3	-0.380	7.57	1.61E-04	0.047
PREDICTED: similar to MHC class II antigen beta chain (LOC641240)	LOC641240	-0.373	9.06	3.70E-04	0.072
cDNA sequence BC028528 (BC028528)	BC028528	-0.363	8.85	9.21E-04	0.097
Growth differentiation factor 10 (Gdf10)	GDF10	-0.359	7.50	3.43E-05	0.028
3'-Phosphoadenosine 5'-phosphosulfate synthase 2 (Papss2)	PAPSS2	-0.355	8.29	2.19E-04	0.055
Yippee-like 3 (Drosophila) (Ypel3)	YPEL3	-0.354	11.52	1.46E-04	0.045
Midkine (Mdk), transcript variant 3	MDK	-0.340	7.59	1.04E-04	0.039
Leucyl-tRNA synthetase (Lars)	LARS	-0.335	8.78	8.56E-05	0.038
Cytochrome P450, family 27, subfamily a, polypeptide 1 (Cyp27a1)	CYP27A1	-0.331	8.75	6.08E-04	0.087
Multimerin 2 (Mmrn2)	MMRN2	-0.321	9.22	1.21E-04	0.040
Insulin-like growth factor binding protein 6 (Igfbp6)	IGFBP6	-0.304	7.68	6.99E-04	0.092
PREDICTED: similar to HLA-G protein (LOC674135), misc RNA	LOC674135	-0.293	8.70	4.65E-04	0.081

	WDFY1	-0.293	7.79	1.12E-04	0.039
Secreted acidic cysteine rich glycoprotein (Sparc)	SPARC	-0.291	10.67	6.25E-04	0.087
Aagrin (Agrn)	AGRN	-0.290	9.64	2.15E-04	0.055
PREDICTED: sterile alpha motif domain containing 9-like, transcript variant 1 (Samd9l)	SAMD9L	-0.289	9.22	9.77E-04	0.099
Bromodomain containing 2 (Brd2), transcript variant 1	BRD2	-0.287	9.14	8.57E-04	0.096
PREDICTED: glutaminase, transcript variant 3 (Gls)	GLS	-0.281	8.25	6.56E-04	0.088
Integrin, beta-like 1 (Itgbl1)	ITGBL1	-0.279	8.22	9.24E-04	0.097
	CCDC3	-0.279	7.29	8.99E-04	0.096
Transforming growth factor, beta receptor III (Tgfbr3)	TGFBR3	-0.277	8.32	3.20E-04	0.067
Progressive ankylosis (Ank)	ANK	-0.275	10.20	5.07E-04	0.081
	6330403M23RIK	-0.272	9.22	5.39E-04	0.084
PREDICTED: RIKEN cDNA 1810013L24 gene (1810013L24Rik)	1810013L24RIK	-0.270	8.36	5.13E-04	0.081
PREDICTED: golgi autoantigen, golgin subfamily b, macrogolgin 1, transcript variant 9 (Golgb1)	GOLGB1	-0.268	8.17	8.04E-04	0.096
Cadherin 5 (Cdh5)	CDH5	-0.265	7.98	7.67E-04	0.096
Matrix metallopeptidase 2 (Mmp2)	MMP2	-0.264	9.93	2.55E-04	0.061
	4930533K18RIK	-0.262	8.22	6.39E-04	0.087
	1110046J11RIK	-0.261	11.35	8.01E-04	0.096
Ankyrin repeat domain 12 (Ankrd12)	ANKRD12	-0.259	7.71	5.01E-04	0.081
ATP-binding cassette transporter sub-family A member 9 (Abca9)	ABCA9	-0.259	7.73	3.64E-04	0.072
	LOC98434	-0.253	8.58	5.76E-04	0.086
Lymphocyte antigen 6 complex, locus E (Ly6e)	LY6E	-0.252	8.68	8.70E-04	0.096
	CD8B	-0.249	9.21	9.16E-04	0.097
SWI/SNF related, matrix associated, actin dependent regulator of chromatin, subfamily c, member 2 (Smarcc2)	SMARCC2	-0.246	7.82	8.85E-04	0.096
Inositol polyphosphate phosphatase-like 1 (Inppl1)	INPPL1	-0.240	10.74	5.88E-04	0.087
AHNAK nucleoprotein 2 (Ahnak2)	AHNAK2	-0.239	7.73	6.19E-04	0.087
3-Hydroxybutyrate dehydrogenase, type 2 (Bdh2)	BDH2	-0.232	7.23	7.32E-04	0.093
	PLD1	-0.228	7.66	8.24E-04	0.096
	1700041B20RIK	-0.218	8.19	8.77E-04	0.096

FIGURE S1: Time course of contractile dysfunction in response to DOX treatment

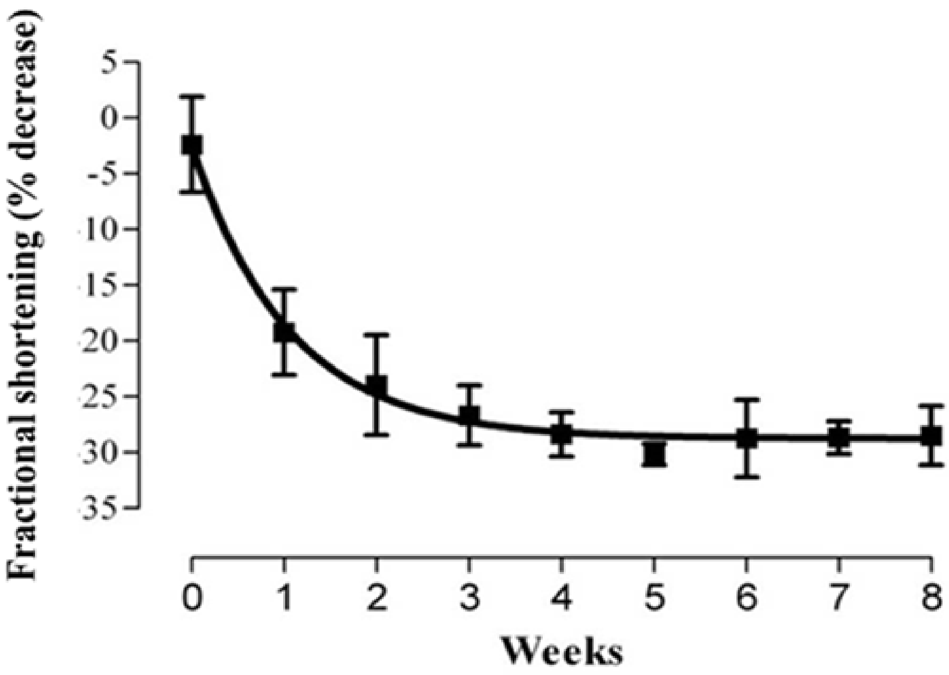

WT mice were administered saline or DOX (4mg/kg) by 3 weekly intraperitoneal (i.p.) injections and fractional shortening quantified at weekly intervals by echocardiography (n=9). Data are shown as mean±SEM and represent % decrease compared to WT control.

FIGURE S2: Effect of DOX on LV NOS isoenzyme and antioxidant gene mRNA expression

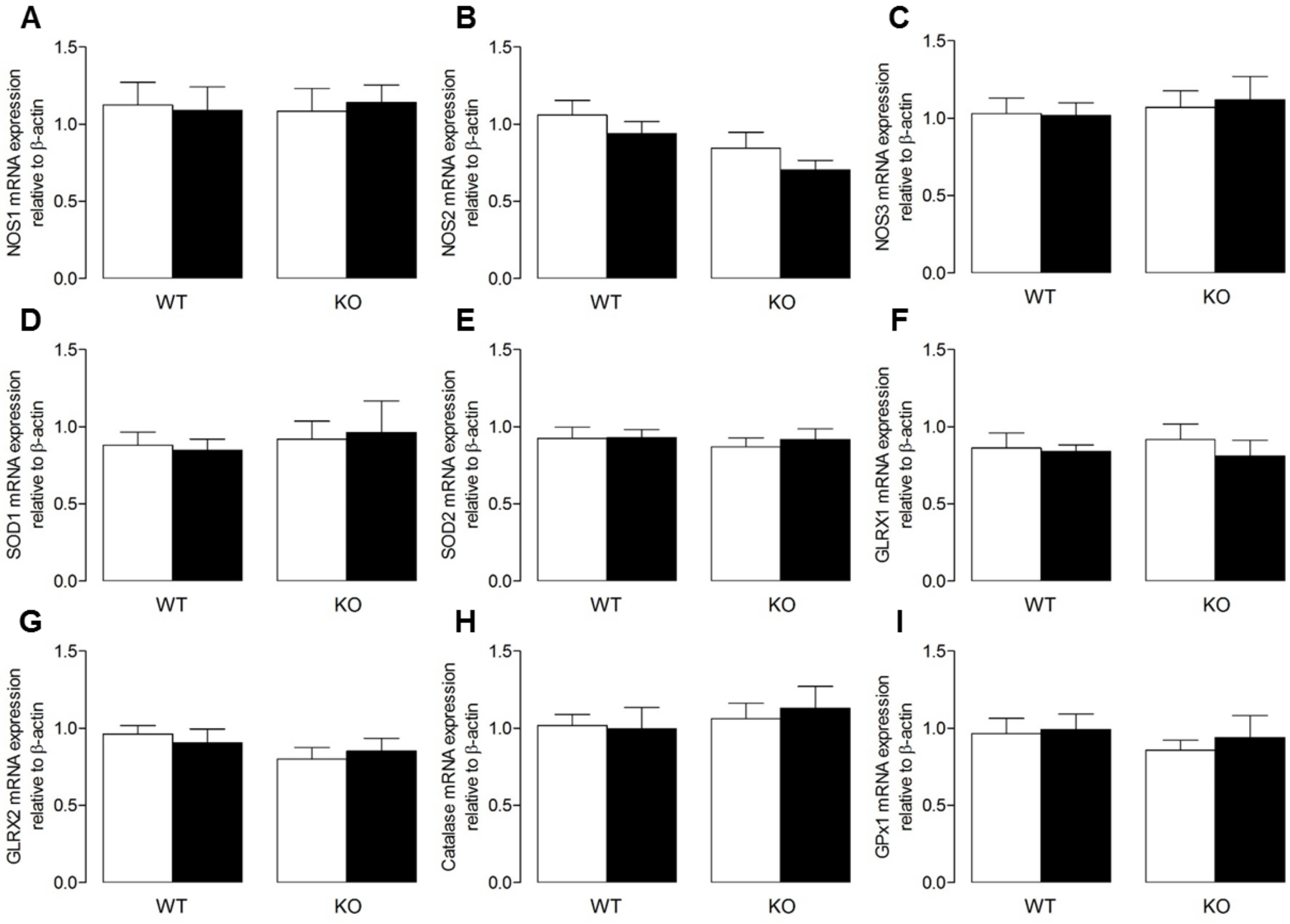

mRNA expression of (A) NOS1 (n=11-15), (B) NOS2 (n=12-13), (C) NOS3 (n=6-9), (D), superoxide dismutase 1 (SOD1, n=6-9), (E) SOD2 (n=7-9), (F), glutaredoxin 1 (GLRX1, n=6-8), (G) GLRX2 (n=11-13), (H) catalase (n=7-8), and (I) glutathione peroxidase (GPx1, n=10-13) by real-time RT-PCR. Data (Control □, DOX ■) are shown as mean (±SEM) and analyses performed using a two-factor ANOVA. P=NS.

FIGURE S3: Quality control assessment of Illumina MouseWG-6 v2.0 microarray

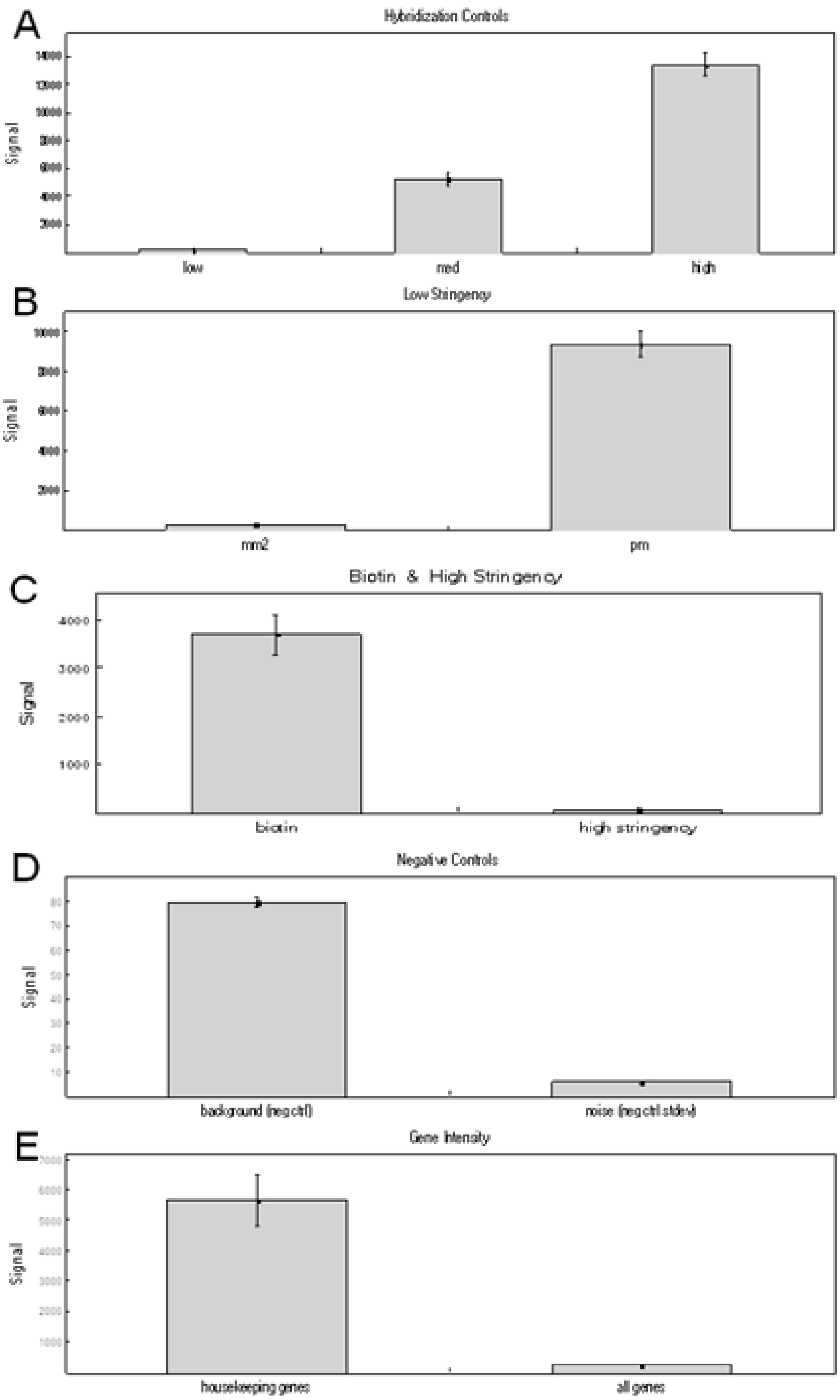

cDNA was generated from WT and Nox2$^{-/-}$ DOX-treated mouse LV (n=3) and used in evaluation of signal intensity using probes for: (A) Hybridisation; (B) Mismatch (mm2) and perfect match (pm); (C) biotin; (D) background and noise and (E) gene intensity. Signal was measured as fluorescence and expressed in arbitrary units. Bar graphs were generated by Cambridge Genomic Services.

FIGURE S4: Comparison of normalised transcript data within samples from WT and Nox2$^{-/-}$ mice.

A

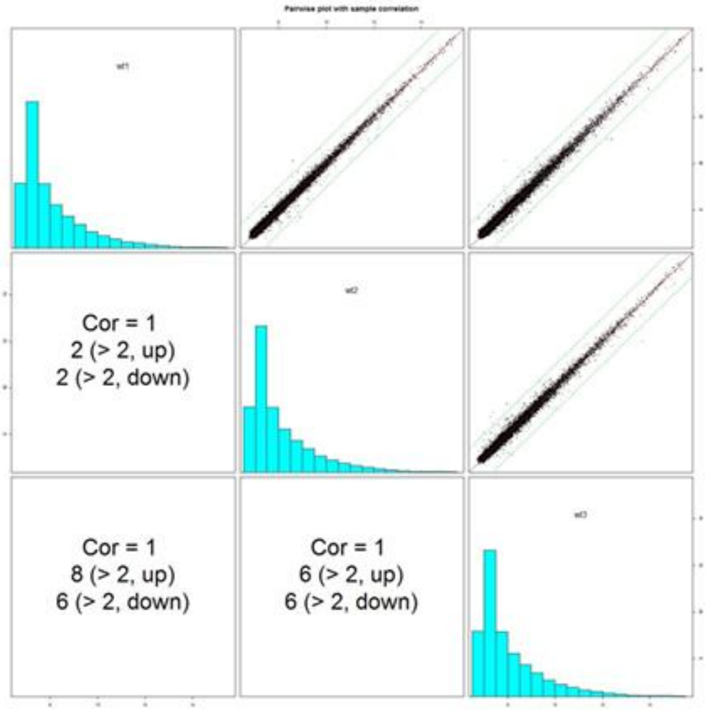

B

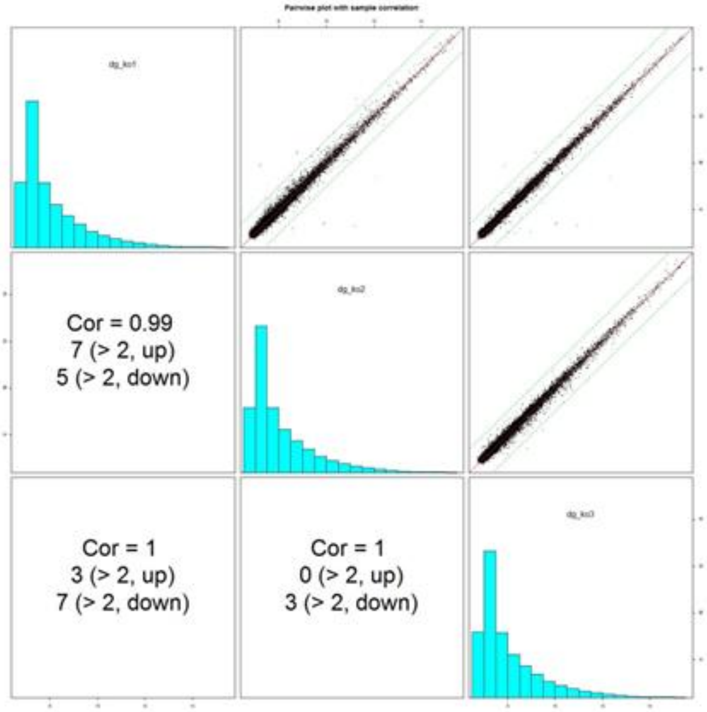

A correlation coefficient (Cor) was generated between each sample in the (A) WT group and (B) Nox2$^{-/-}$ group. Graphs were generated in the *R* programme using the *lumi* package by Cambridge Genomics Services.

FIGURE S5: Concentration responses for optimisation of experimental conditions for the ApoTox-Glo Triplex assay in HL-1 cardiomyocytes

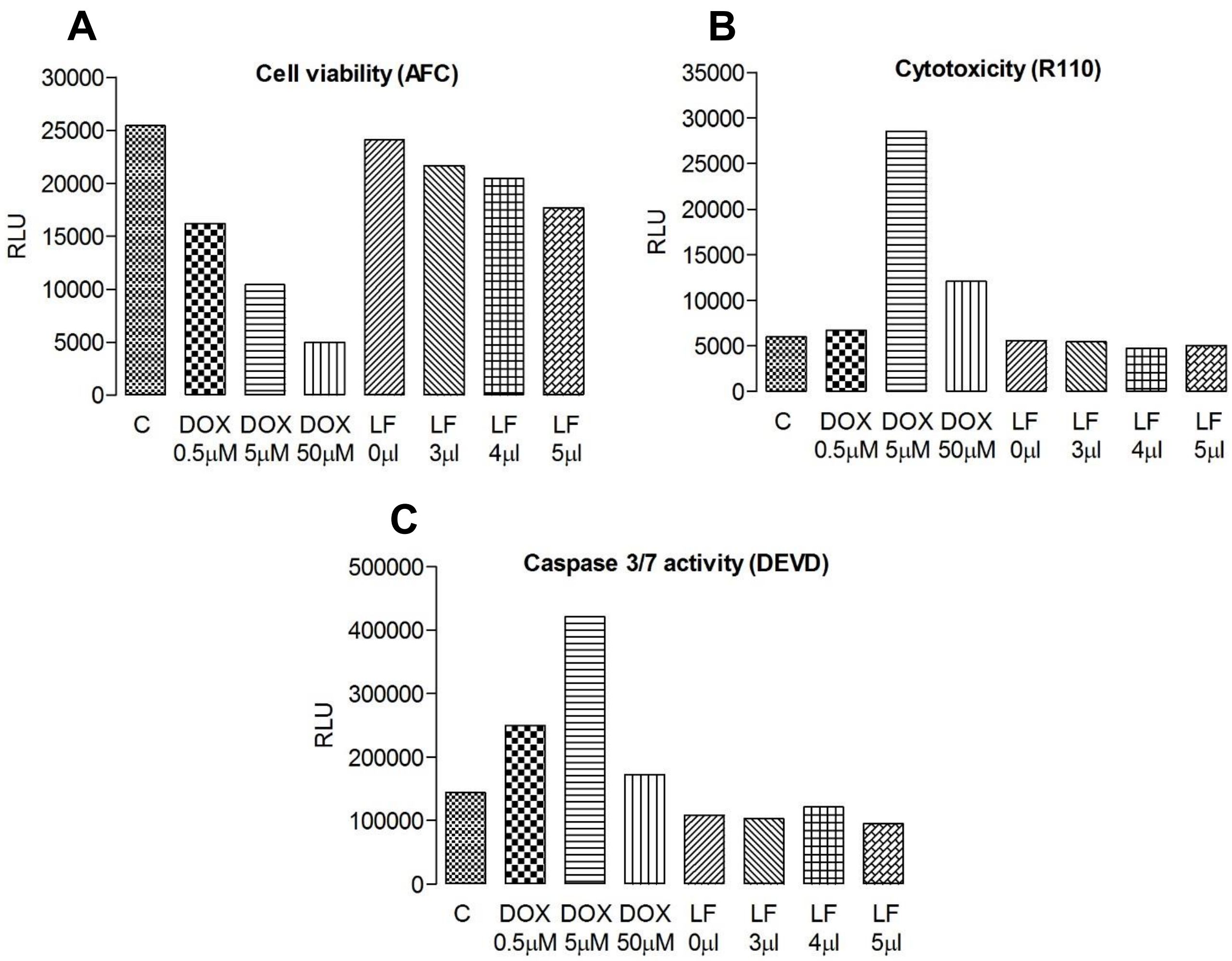

HL-1 cardiomyocytes (seeded 20000 cells/well) in a 96 well plate format were incubated in normal medium (Control, C) or with increasing concentrations of DOX (0.5µM, 5µM, 50µM), or varying volumes (0-5µl/100µl total well volume) of LF2000 (LF) for 24h prior to detection by fluorescence. (A) Cell viability (aminofluorocoumarin, AFC) and (B) cytotoxicity (rhodamine 110, R110), and by luminescence (C) Caspase 3/7 activity (cleavage of DEVD). Data are shown as average value (n=2, 8-10 replicates in each). RLU, relative light units.
